# Comprehensive Analyses of the Expression, Genetic Alteration, Prognosis Significance, and Interaction Networks of m^6^A Regulators Across Human Cancers

**DOI:** 10.3389/fgene.2021.771853

**Published:** 2021-12-23

**Authors:** Xiujuan Shi, Jieping Zhang, Yuxiong Jiang, Chen Zhang, Xiaoli Luo, Jiawen Wu, Jue Li

**Affiliations:** ^1^ Clinical Research Center for Mental Disorders, Shanghai Pudong New Area Mental Health Center, School of Medicine, Tongji University, Shanghai, China; ^2^ School of Medicine, Tongji University, Shanghai, China; ^3^ Shanghai East Hospital, Tongji University School of Medicine, Shanghai, China

**Keywords:** m^6^A methylation, bioinformatics, TCGA, cancer, comprehensive analyses

## Abstract

Accumulating lines of evidence indicate that the deregulation of m^6^A is involved in various cancer types. The m^6^A RNA methylation is modulated by m^6^A methyltransferases, demethylases, and reader proteins. Although the aberrant expression of m^6^A RNA methylation contributes to the development and progression of multiple cancer types, the roles of m^6^A regulators across numerous types of cancers remain largely unknown. Here, we comprehensively investigated the expression, genetic alteration, and prognosis significance of 20 commonly studied m^6^A regulators across diverse cancer types using TCGA datasets *via* bioinformatic analyses. The results revealed that the m^6^A regulators exhibited widespread dysregulation, genetic alteration, and the modulation of oncogenic pathways across TCGA cancer types. In addition, most of the m^6^A regulators were closely relevant with significant prognosis in many cancer types. Furthermore, we also constructed the protein–protein interacting network of the 20 m^6^A regulators, and a more complex interacting regulatory network including m^6^A regulators and their corresponding interacting factors. Besides, the networks between m^6^A regulators and their upstream regulators such as miRNAs or transcriptional factors were further constructed in this study. Finally, the possible chemicals targeting each m^6^A regulator were obtained by bioinformatics analysis and the m^6^A regulators–potential drugs network was further constructed. Taken together, the comprehensive analyses of m^6^A regulators might provide novel insights into the m^6^A regulators’ roles across cancer types and shed light on their potential molecular mechanisms as well as help develop new therapy approaches for cancers.

## Introduction

A variety of biological processes are orchestrated by post-transcriptional modifications including RNA modifications (Zhao et al., 2017). As the most common type of RNA methylation modifications, N6-methyladenosine (m^6^A), first unraveled in 1970s, modulates the corresponding target RNAs *via* influencing RNA translation, degradation, splicing, folding, or stability (He and He, 2021). Although studies had revealed that one to two m^6^A residues were found in an average of one thousand nucleotides, nearby the 3′ untranslated region (UTR), stop codon as well as long internal exon might exhibit the relatively richer m^6^A in mRNAs (Meyer et al., 2012). In addition to mRNAs, m^6^A RNA methylation was also found to be distributed in other RNAs, such as ribosomal RNA (rRNA) and RNAs of bacteria and viruses (Ma et al., 2019).

The regulators involved in modulating m^6^A methylation include three types of proteins called “writer”, “eraser”, and “reader”, respectively (Zaccara et al., 2019). The writers consisting of m^6^A methyltransferases such as METTL3, METTL14, and their corresponding cofactors like RBM15 and WTAP exhibit in cellular nuclei and increase the m^6^A levels (Meyer and Jaffrey, 2017). On the contrary, the erasers, also being discovered in the cellular nuclei, are m^6^A demethylase enzymes such as FTO and ALKBH5, which remove the m^6^A and thus result in reducing the m^6^A levels (Zhang et al., 2017). Moreover, the readers such as IGF2BP1 and RBMX, distributing in both cellular nuclei and cytoplasm, can decode the m^6^A methylation information *via* binding to the m^6^A sites and further initiate the different downstream signals (Sun et al., 2019). The processes of m^6^A methylation are reversible and dynamic, which are homeostatically modulated by these writers, erasers, and readers (Chen et al., 2019).

Since the m^6^A regulators acted as crucial roles in a variety of biological processes, the abnormalities of m^6^A methylation might lead to multiple kinds of diseases including neuronal diseases, diabetes, immunological disorders, liver metabolic disorders, and numerus cancer types (He et al., 2019). For example, recent studies had demonstrated that the decreased RNA methylation of critical genes in β-cell markedly contributed to the pathophysiology of human T2D (De Jesus et al., 2019). Additionally, METTL3 was found to have dramatical overexpression in hepatocellular carcinoma (HCC), and the depletion of METTL3 contributed to the significant suppression of the HCC growth and metastasis (Chen et al., 2018). Besides, findings had uncovered that YTHDF2, an m^6^A reader, was markedly upregulated in human acute myeloid leukemia (AML), and targeting YTHDF2 might compromise the cancer stem cells in AML (Paris et al., 2019).

Although the m^6^A methylation has been identified as the most abundant modification of RNAs, and served as crucial regulators in diverse biological processes and diseases including numerous types of cancers, the relevant factors involved in that modification are still not completely discovered, and their associated molecular mechanisms, expression, and interacting networks remain unclear. Therefore, in the present study, we comprehensively investigated the expression, genetic alteration, and prognosis significance of 20 commonly studied m^6^A regulators across diverse cancer types using TCGA *via* bioinformatic analyses. In addition, we also constructed the networks between m^6^A regulators and potential chemical drugs, miRNAs, or upstream transcriptional factors. These comprehensive analyses of m^6^A regulators might provide novel understanding of these m^6^A regulators’ roles across cancer types and shed light on their potential molecular mechanisms in cancers as well as helping developing new therapy approaches for cancers.

## Materials and Methods

### The Gene Expression and Methylation Analyses of m^6^A Regulators

The expression of the gene set (the 20 m^6^A methylation regulators) and m^6^A regulators’ interacting proteins across diverse cancer types based on TCGA data was analyzed using the GSCALite database (Liu et al., 2018). Besides, we also analyzed the expression of 20 m^6^A methylation regulators through R software package using microarray data (GSE11969, GSE63898, GSE37182, GSE22820, GSE54129, GSE53757, GSE23036, GSE33630, and GSE11024) from the Gene Expression Omnibus (GEO) datasets. The heatmaps of these GEO data were displayed by the R software package pheatmap. The expression of IGF2BP1, IGF2BP2, IGF2BP3, SP1, ELK1, and EGR1 across diverse TCGA cancer types was analyzed using the UALCAN database (Chandrashekar et al., 2017). In addition, the methylation of the gene set, and the correlation between the methylation and m^6^A methylation regulators’ gene expression were also analyzed using GSCALite database.

### The Genetic Alteration Analyses of the m^6^A Regulators

The single nucleotide variations (SNVs) and copy number variations (CNVs) of the m^6^A regulators across cancer types were analyzed by the GSCALite database using TCGA data. The SNV-oncoplot and CNV-percent-profile (CNV pie plots) were also generated by GSCALite database. In addition, the bubble plots describing the correlation between CNV and m^6^A methylation regulators’ mRNA expression were generated by GSCALite database based on TCGA data. Besides, the genetic alterations of the 20 m^6^A regulators were also analyzed by cBioportal database (Cerami et al., 2012).

### The Oncogenic Pathway Analyses of the m^6^A Regulators and Protein–Protein Interaction Network Construction

The m^6^A methylation regulators-related oncogenic pathways were analyzed by GSCALite database. The pathway activity pie plots, and the interaction map of genes and pathways were also generated using the GSCALite database. The protein–protein interaction (PPI) networks were generated by STRING database (Szklarczyk et al., 2019).

### The Overall Survivals Analyses

The overall survivals of the m^6^A methylation regulators across cancer types were analyzed by the GSCALite database. The overall survivals of the m^6^A regulators in kidney renal clear cell carcinoma (KIRC) were evaluated by the GEPIA database based on TCGA data.

### The m^6^A Regulators–Drug Interacting Network Construction

The potential chemicals targeting each m^6^A regulator were obtained by applying the comparative toxicogenomics database (CTD) (Davis et al., 2021). Thereafter, the chemicals and their corresponding m^6^A regulator were inputted into Cytoscape software (Shannon et al., 2003) to generate the m^6^A regulators–drug interacting network.

### The Generation of the MiRNAs–m^6^A Regulators Network

First, the potential miRNAs targeting each m^6^A regulator were predicted by miRDB (Chen and Wang, 2020), targetScan (Agarwal et al., 2015), and starbase (Li et al., 2014). Subsequently, the overlapping miRNAs (commonly expressed in the prediction of miRDB, targetScan, and starbase) were obtained using the VENNY 2.1 database (https://bioinfogp.cnb.csic.es/tools/venny/index.html). Then, the Cytoscape software was utilized for generating the miRNAs–m^6^A regulators networks.

### Constructing the Transcription Factors–m^6^A Regulators Network

The potential TFs targeting each group of m^6^A regulators (writers, erases, and readers) were obtained by KnockTF database (Feng et al., 2020). The erasers–TFs interacting network, writers–TFs interacting network, and readers–TFs interacting network were also generated by KnockTF database. In addition, the top 25 potential TFs were achieved exhibited by doughnut plots using FunRich software (Pathan et al., 2015).

### The Interacting Networks Construction and Gene Ontology and Biological Pathway Analyses

The m^6^A regulators’ interacting proteins were obtained by FunRich software, and the interacting regulatory network including m^6^A regulators and their corresponding interacting proteins was then constructed by FunRich software. The FunRich software was also utilized for investigating the GO analyses and biological pathways of the m^6^A regulators interacting proteins. The column diagrams (exhibiting CC: cellular component; MF: molecular function; BP: biological process) and doughnut plots (exhibiting biological pathways) were also generated using FunRich software.

## Results

### The Expression of m^6^A Methylation Regulators Across Cancer Types

**FIGURE 1:**
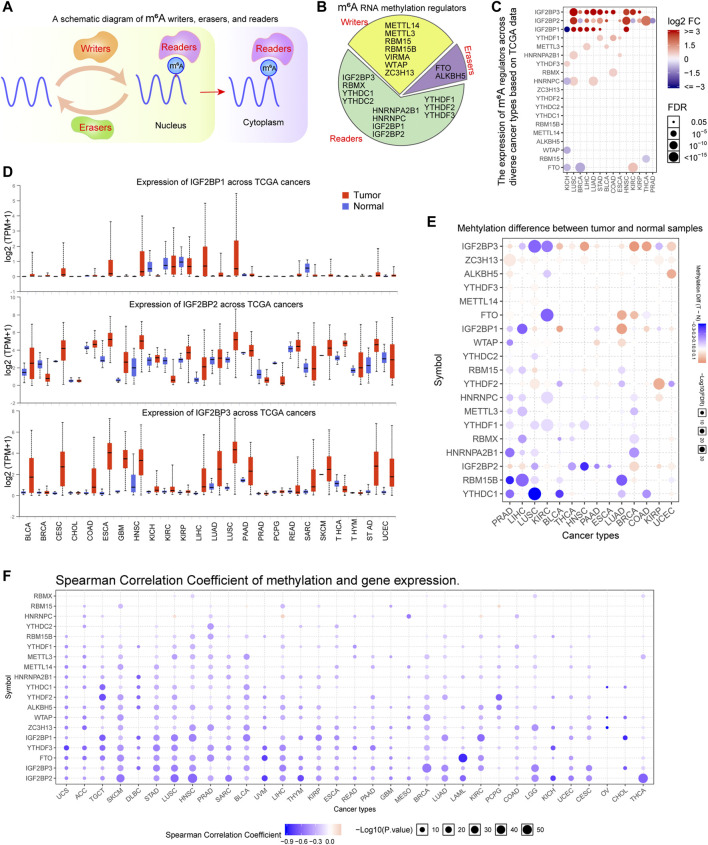
The expression analyses of m^6^A regulators in TCGA cancer types. **(A)** A schematic diagram of m^6^A regulators in cells. **(B)** The m^6^A regulators are divided into writers, erasers, and readers. **(C)** The GSCALite database revealed the expressing levels of the 20 m^6^A regulators across diverse cancer types. **(D)** Details expressing situations of IGF2BP1, IGF2BP2, and IGF2BP3 were evaluated by applying the UALCAN algorithm. **(E)** The methylation difference analyzed by GSCALite between tumor and normal samples of m^6^A regulators across TCGA cancer types. **(F)** The correlation between the methylation and m^6^A methylation regulators’ gene expression were analyzed using GSCALite database.

The m^6^A methylation regulators could be clarified into three types: writers, erasers, and readers (Figure 1A). The reports relevant with m^6^A methylation regulators in recent years were reviewed and a total of 20 genes (writers: 7; erasers: 2; readers: 11) were included in this study (Figure 1B). Next, we sought to evaluate the expressing levels of these 20 m^6^A methylation regulators across diverse cancer types using TCGA datasets. By searching the GSCALite database, we found that the expressions of many m^6^A methylation regulators (especially IGF2BP1, IGF2BP2, and IGF2BP3) were changed across multiple cancer types (Figure 1C). Furthermore, the detail expressing situations of IGF2BP1, IGF2BP2, and IGF2BP3 were evaluated by applying UALCAN algorithm. The results revealed that the levels of IGF2BP1, particularly IGF2BP2 and IGF2BP3, were remarkably upregulated in many cancer types such as bladder urothelial carcinoma (BLCA), liver hepatocellular carcinoma (LIHC), lung adenocarcinoma (LUAD), and lung squamous cell carcinoma (LUSC) (Figure 1D). In addition, to further verify the above results using TCGA data, we also analyzed the expression of 20 m^6^A regulators using microarray data from GEO datasets in many cancer types including LUAD, LUSC, LIHC, colon adenocarcinoma (COAD), breast invasive carcinoma (BRCA), stomach adenocarcinoma (STAD), kidney renal clear cell carcinoma (KIRC), head and neck squamous cell carcinoma (HNSC), thyroid carcinoma (THCA), and kidney chromophobe (KICH). The results revealed that the expression of many m^6^A regulators including IGF2BP1, IGF2BP2, and IGF2BP3 were notably changed, which was consistent with the results from TCGA analysis (Supplementary Figure S1). Given that the methylation of genes was able to influence genes expression, we next attempted to investigate the methylation of the 20 m^6^A methylation regulators across cancer types. Through searching GSCALite database, we found that the majority of the 20 m^6^A regulators’ methylation was lower in the tumor samples than that of the normal control samples in prostate adenocarcinoma (PRAD), LIHC, LUSC, KIRC, BLCA, and THCA (Figure 1E). Besides, the correlation between the methylation and m^6^A methylation regulators’ gene expression was further analyzed using the GSCALite database. According to the data, we found that most of these m^6^A regulators’ expression was negatively correlated with the methylation across diverse cancer types, which was consistent with the above finding that the majority of the 20 m^6^A regulators’ methylation was downregulated in the tumor samples of many cancer types (Figure 1F). Overall, our data suggested that the expression and methylation of the 20 m^6^A regulators were remarkable dysregulation across many cancer types.

### Genetic Alterations of m^6^A Regulators Across Cancer Types

**FIGURE 2:**
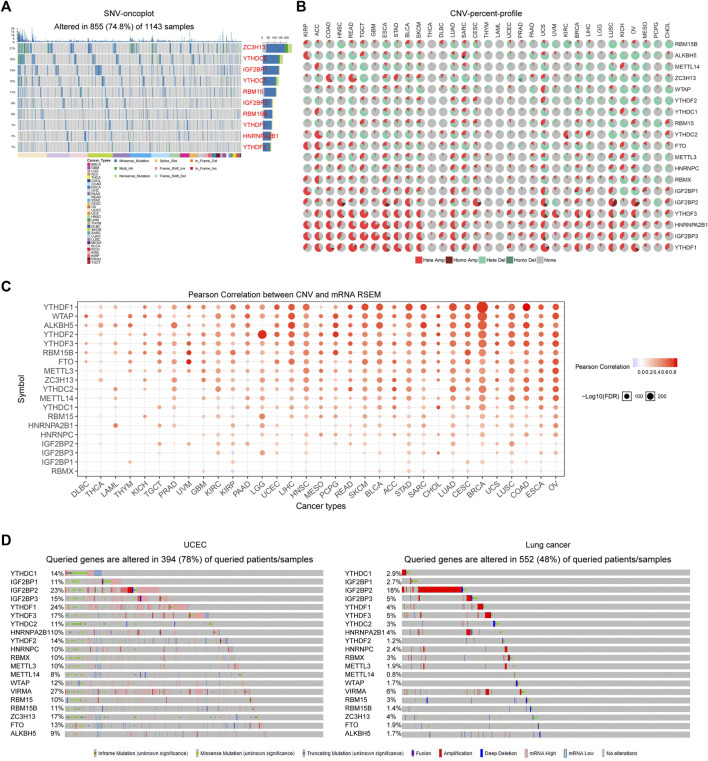
The genetic alterations of m6A regulators. **(A)** The SNVs of the m^6^A regulators in TCGA samples across cancer types. The waterfall plots presented the top ten SNVs-changed genes. **(B)** The CNV pie plots revealed the CNVs alteration frequency of the 20 m^6^A regulators across diverse cancer types. **(C)** The bubble plots from GSCALite database showed that the mRNA expression of the m^6^A regulators was positively correlated with their corresponding CNVs across most cancer types. **(D)** The genetic alterations of the 20 m^6^A regulators in UCEC and lung cancer using cBioportal analyses.

Considering that the alteration of the genome might always affect the gene expression, we next attempted to explore the genetic alterations including single nucleotide variations (SNVs) and copy number variations (CNVs) of the m^6^A regulators across cancer types. First, the GSCALite database was utilized for analyzing the SNVs of the 20 m^6^A regulators. The results suggested that the SNVs of the 20 m^6^A regulators altered in 74.8% TCGA samples across cancer types, and the waterfall plots presented the top ten SNVs-changed genes, such ZC3H13, YTHDC2, and IGF2BP1 (Figure 2A). Thereafter, we sought to investigate the CNVs alteration frequency for the 20 m^6^A regulators using the GSCALite database. The CNV pie plots revealed that several readers (YTHDF1, YTHDF3, IGF2BP1, IGF2BP2, IGF2BP3, and HNRNPA2B1) exhibited very high percentages of heterozygous CNVs, particularly amplification (Hete Amp) in multiple cancer types, while genes like RBM15B, ALKBH5, METTL14, ZC3H13, and WTAP had high percentages of heterozygous CNVs with depletion (Hete Del) (Figure 2B). Afterwards, we attempted to explore whether these m^6^A regulators’ CNVs alterations were able to influence the expression of their mRNA expression. The bubble plots from GSCALite database demonstrated that the mRNA expression of the majority of the m^6^A regulators was positively correlated with their corresponding CNVs across most cancer types, which indicated that CNVs alterations could remarkably promote m^6^A regulators’ expression (Figure 2C). In addition, the genetic alterations of the 20 m^6^A regulators in TCGA cancer types were also investigated using cBioportal database. The data suggested that the genetic alterations of the m^6^A regulators were remarkably high in tumor specimens of many cancer types, particularly uterine corpus endometrial carcinoma (UCEC; with 78% genetic alterations) and lung cancer (with 48% genetic alterations) (Figure 2D). Taken together, these results revealed that the m^6^A regulators exhibited widespread genetic alterations across cancer types, and these genetic alterations could significantly affect their expression.

### The Analyses of the Oncogenic Pathways Relevant With the m^6^A Regulators

**FIGURE 3:**
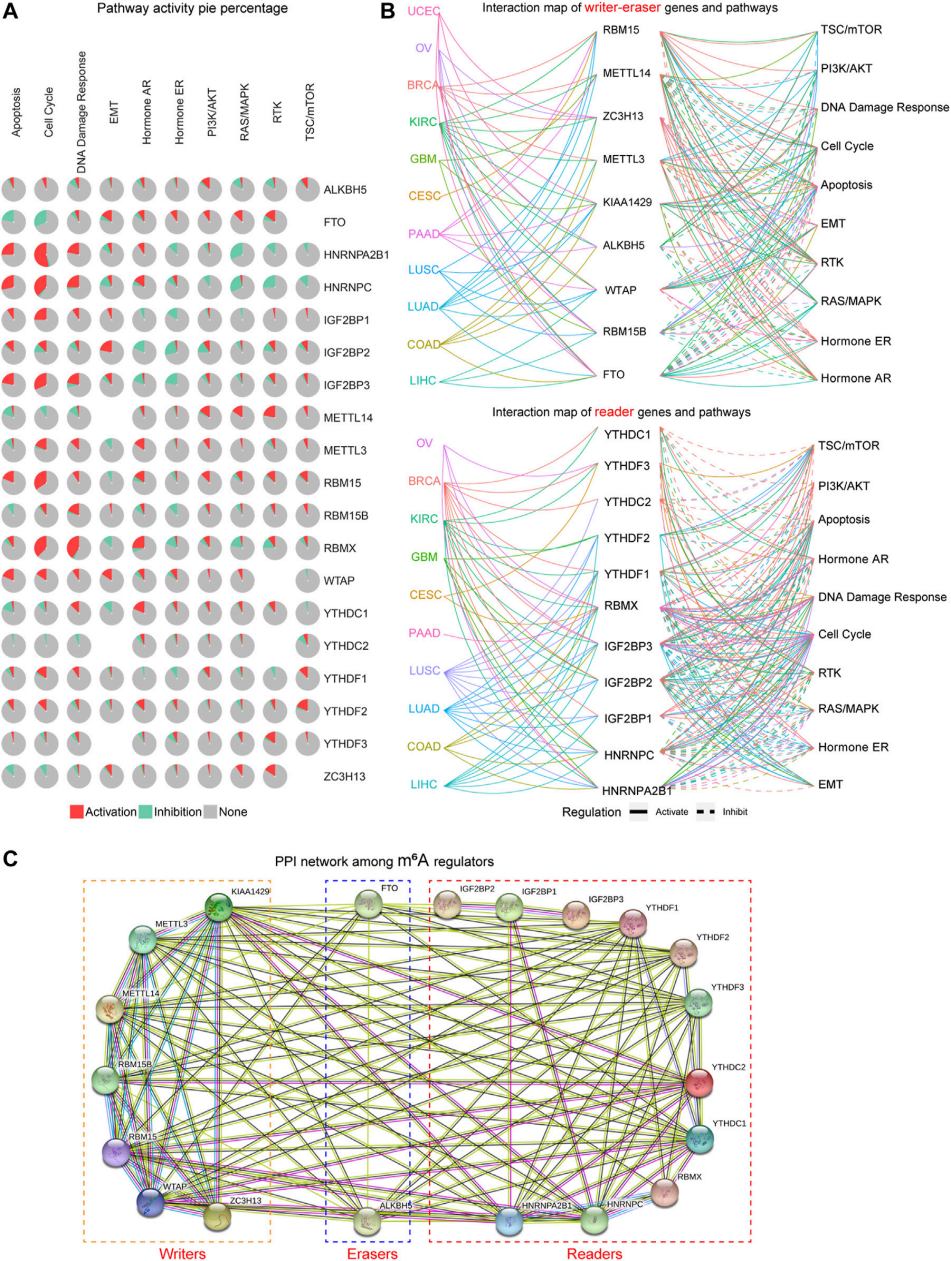
The oncogenic pathways related to the m^6^A regulators. **(A)** The pathway pie plots analysis of m^6^A regulators from the GSCALite database. **(B)** The interaction map of m^6^A regulators and pathways in numerous cancer types using GSCALite database. **(C)** The protein–protein interaction (PPI) network of the 20 m^6^A regulators was constructed by STRING database.

Next, we attempted to investigate whether these m^6^A regulators were associated with oncogenic pathways. According to the results of the pathway pie plots from GSCALite database, we found that HNRNPA2B1, HNRNPC, IGF2BP1, IGF2BP3, RBM15, and RBMX were markedly related with the activation of the cell cycle (Figure 3A). In addition, the pathway pie plots also showed that FTO was relevant with the inhibition of apoptosis and cell cycle; HNRNPA2B1 and HNRNPC were significantly correlated with the inhibition of the RAS/MAPK pathway; RBMX was also related with the activation of DNA damage response pathway (Figure 3A). Besides, the 20 m^6^A regulators were divided into two groups (writer–eraser genes and reader genes) to respectively construct the interaction map of genes and pathways using the GSCALite database, and the results further confirmed the above findings that many m^6^A regulators were associated with the activation or inhibition of these famous cancer-related pathways across TCGA cancer types (Figure 3B). Furthermore, considering that genes always exerted their functions *via* interacting with other genes, we thereby next sought to investigate the interaction among these writers, erasers, and readers. The protein–protein interaction (PPI) network of the 20 m^6^A regulators was constructed by the STRING database, and the PPI network demonstrated that the m^6^A regulators interacted with each other with very high frequency, which indicated that the m^6^A methylation in cancers might be regulated by collaboration among writers, erasers, and readers (Figure 3C). Collectively, these data validated that the m^6^A regulators could modulate the oncogenic pathways *via* collaboration.

### Prognosis Significance of the m^6^A Regulators Across Cancer Types

**FIGURE 4:**
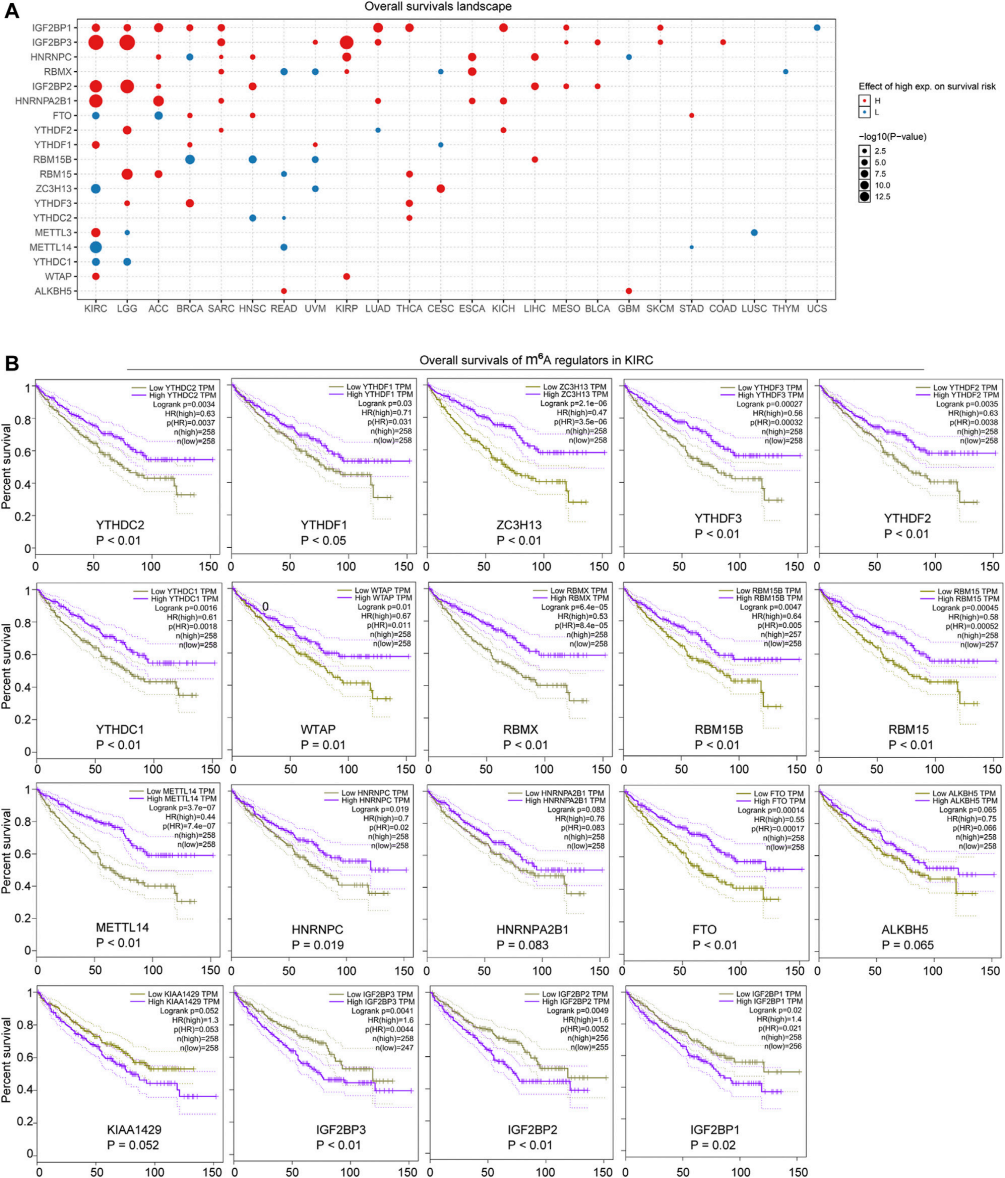
Prognosis significance of the m^6^A regulators across cancer types. **(A)** Overall survivals landscape of the m^6^A regulators across cancer types was generated by GSCALite database. **(B)** GEPIA analyzed the overall survivals of the m^6^A regulators in KIRC.

Since the m^6^A regulators were dysregulated in many cancer types and several of them were closely relevant with oncogenic pathways, we next sought to explore whether the aberrant expression of the m^6^A regulators was associated with prognosis significance. After inputting the gene set of the m^6^A regulators into the GSCALite database, we found that most of the m^6^A regulators were associated with overall survivals across TCGA cancer types (Figure 4A). Particularly, more than half of the 20 m^6^A regulators were notably correlated with poor or good prognosis in multiple cancer types, such as KIRC, brain lower grade glioma (LGG), adrenocortical carcinoma (ACC), breast invasive carcinoma (BRCA), and sarcoma (SARC). Therefore, we next attempted to investigate the detailed overall survivals of the m^6^A regulators (high or low expression) in KIRC. The overall survivals of the 20 m^6^A regulators in KIRC were analyzed by applying the GEPIA database. The results validated that 19 of the 20 m^6^A regulators were dramatically correlated with significantly good or poor prognosis (Figure 4B). Especially, high expression of all the erasers (FTO and ALKBH5) and most writers (METTL14, RBM15, RBM15B, WTAP, and ZC3H13) and readers (YTHDF1, YTHDF2, YTHDF3, HNRNPA2B1, HNRNPC, YTHDC1, YTHDC2, and RBMX) was significantly with poor prognosis, while the high expression of VIRMA (also named KIAA1429; a writer) and IGF2BPs (IGF2BP1, IGF2BP2, and IGF2BP3; readers) predicted good prognosis (Figure 4B). Therefore, these data revealed that the dysregulation of the m^6^A regulators was remarkably associated with significant prognosis in many cancer types, especially KIRC, which indicated that the aberrant expression of the m^6^A regulators might be a prognostic marker in cancers including KIRC.

### The Construction of the m^6^A Regulators–Potential Drugs Network

**FIGURE 5:**
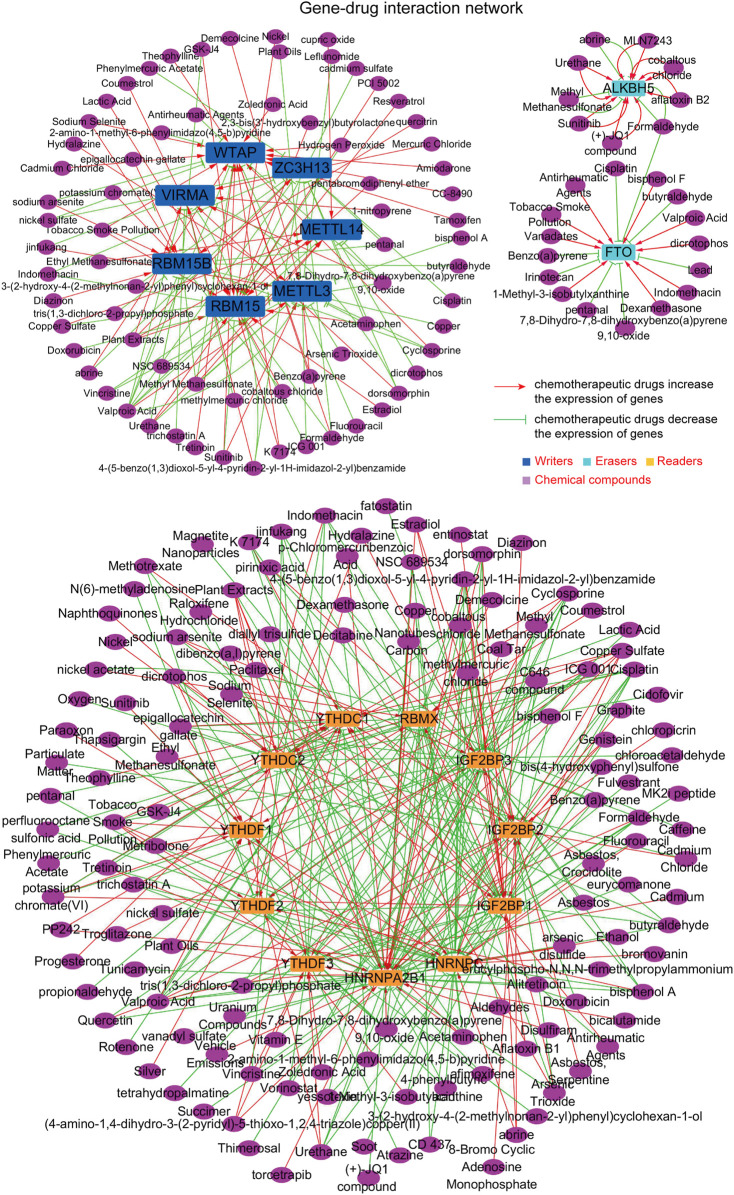
The construction of the m^6^A regulators–potential drugs network. The potential chemicals targeting m^6^A regulators were obtained by the comparative toxicogenomics database (CTD), and the network was generated by Cytoscape software.

Since the above findings revealed that the dysregulation of the m^6^A regulators might be correlated with tumor progression, we next thought to investigate whether there were some potential chemicals that could increase or decrease the expression of the m^6^A regulators. First, the comparative toxicogenomics database (CTD) was utilized for analyzing the possible chemicals targeting each m^6^A regulator. Afterwards, the 20 m^6^A regulators were divided into three groups (writers, erasers, and readers) and we subsequently drew three sub gene–drug interaction networks (writers–drugs interaction network, erasers–drugs interaction network, and readers–drugs interaction network), using Cytoscape software. The m^6^A regulators–potential drugs network is presented in Figure 5, and these chemicals were able to increase or decrease the expression of the m^6^A regulators (Supplementary Table S1). Therefore, the m^6^A regulators–potential drugs network provided benefits for potential drugs discovery to target specific m^6^A regulators.

### The Upstream MiRNAs–m^6^A Regulators Network

**FIGURE 6:**
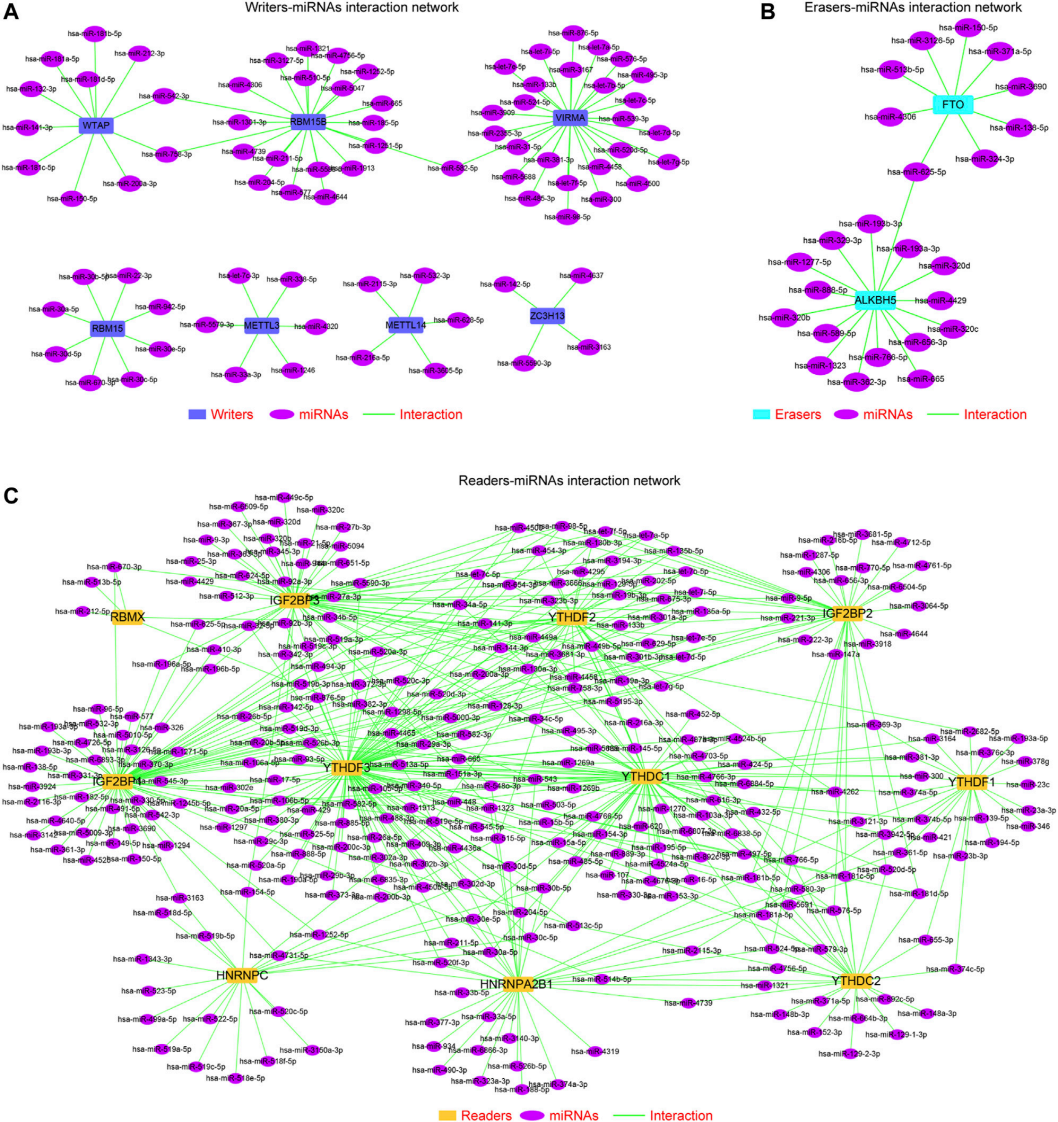
MiRNAs–m^6^A regulators network construction. **(A)** Writers–miRNAs interaction network. **(B)** Erasers–miRNAs interaction network. **(C)** Readers–miRNAs interaction network.

Although our above findings demonstrated that the methylation and genetic alterations were capable to affect the expression of the m^6^A regulators across TCGA cancer types, there might be other factors such as miRNAs that could also contribute to the dysregulation of the m^6^A regulators in cancers. Therefore, we next sought to investigate the potential upstream miRNAs, which were able to target these m^6^A regulators. First, we employed three classical miRNAs predicting databases—miRDB, targetScan, and starbase—to predict the possible miRNAs that could target each m^6^A regulator. Thereafter, the common miRNAs targeting each m^6^A regulator in the three databases were selected. Subsequently, we applied the Cytoscape software to generate the miRNAs–m^6^A regulators networks, including the writers–miRNAs interaction network (Figure 6A), erasers–miRNAs interaction network (Figure 6B), and readers-miRNAs interaction network (Figure 6C). These miRNAs–m^6^A regulators networks provided new supplementary knowledge about the modulation of the m^6^A regulators’ dysregulation across cancer types.

### The Upstream TFs–m^6^A Regulators Networks

**FIGURE 7:**
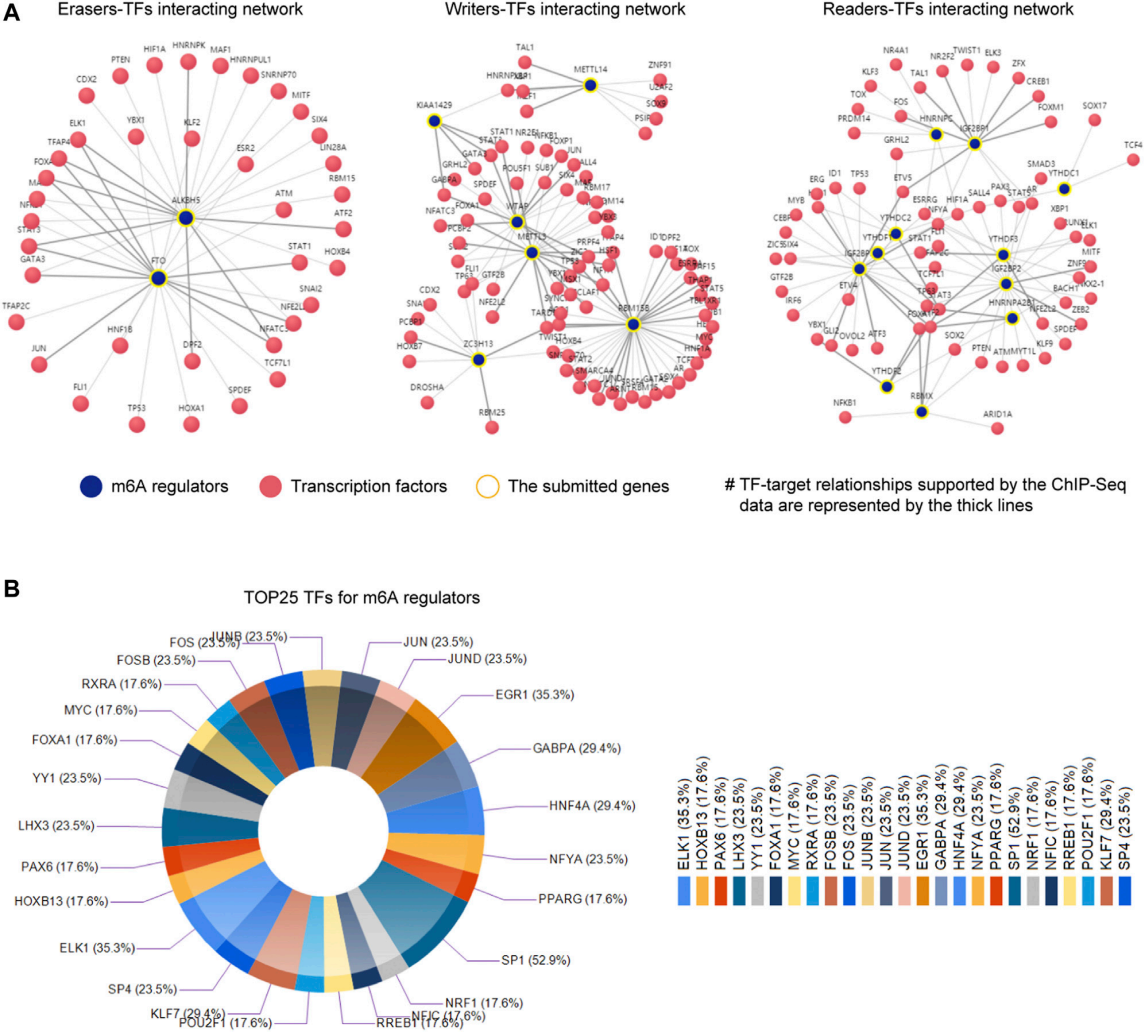
The construction of TFs–m^6^A regulators networks. **(A)** The TFs–m^6^A regulators networks (including three sub-networks: erasers–TFs interacting network, writers–TFs interacting network, and readers–TFs interacting network) were generated by KnockTF software. **(B)** The top 25 potential TFs (ranked by targeting percentages) were achieved and exhibited by doughnut plots using FunRich software.

Besides, the transcription factors (TFs) could also contribute to the dysregulation of m^6^A regulators. Hence, we next sought to uncover the potential TFs that were capable to modulate the expression of the m^6^A regulators. To achieve that, we first utilized an online database, KnockTF, to analyze the possible TFs of the writers, erases, and readers, respectively. We generated the TFs–m^6^A regulators networks (including three sub-networks: erasers–TFs interacting network, writers–TFs interacting network, and readers–TFs interacting network), and they are presented in Figure 7A. The TFs–m^6^A regulators networks indicated that a plethora of TFs might regulate the m^6^A regulators. For example, there were more than 20 TFs that were possibly able to target the promoter of RBM15B. In addition, another software, FunRich, was also utilized for calculating the potential TFs targeting the m^6^A regulators. The top 25 potential TFs (ranked by targeting percentages) were achieved and exhibited by doughnut plots (Figure 7B). The data demonstrated that SP1 were possibly able to modulate more than half of the 20 m^6^A regulators (52.9%), ELK1 regulated 35.3% of the 20 m^6^A regulators, and EGR1 also orchestrated 35.3% of the 20 m^6^A regulators. Indeed, analyses from the UALCAN database revealed that the expressions of SP1, ELK1, and EGR1 were remarkably aberrant in many TCGA cancer types (Supplementary Figure S2). Collectively, these data provided novel insights into the possible molecular mechanisms of the m^6^A regulators’ dysregulation in TCGA cancer types.

### The Interacting Regulatory Network of Each m^6^A Regulators

**FIGURE 8:**
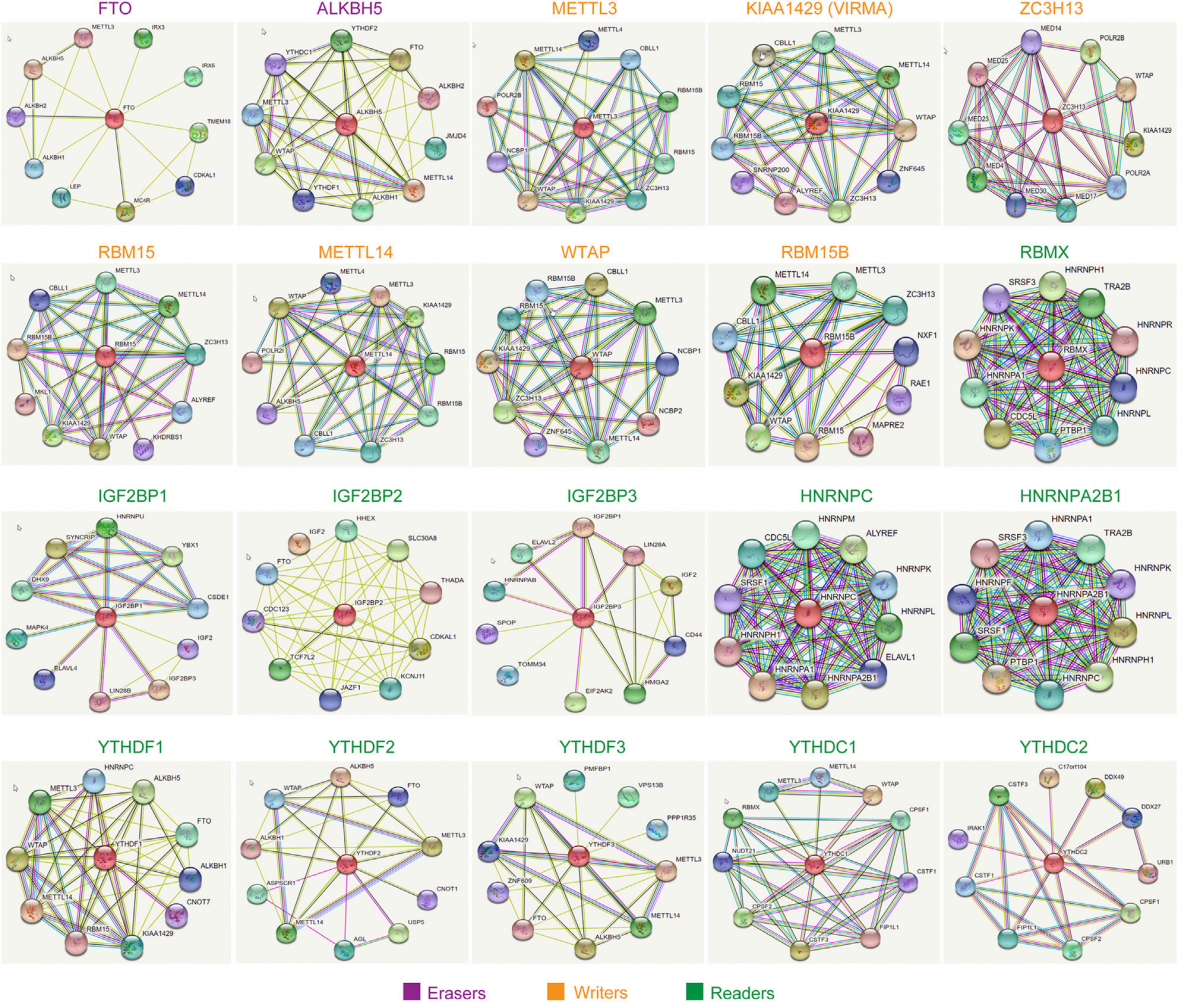
The interacting regulatory network of each m^6^A regulator. The interacting proteins of each eraser, writer, and reader were obtained by using STRING database.

Although we had generated the PPI network of the 20 m^6^A regulators in the above findings, which demonstrated that these 20 m^6^A regulators interacted with each other with very high frequency, the m^6^A regulators exerted their functions and might also collaborate with other factors. Therefore, we next attempted to construct the interacting regulatory network of each m^6^A regulator alone. The interacting proteins of each eraser, writer, and reader were obtained by using the STRING database, and their corresponding interacting networks are presented in Figure 8. The results indicated that every m^6^A regulator had a complex interacting regulatory network, and these interacting networks might provide new insights into how the m^6^A regulators exerted their modulatory functions.

**FIGURE 9:**
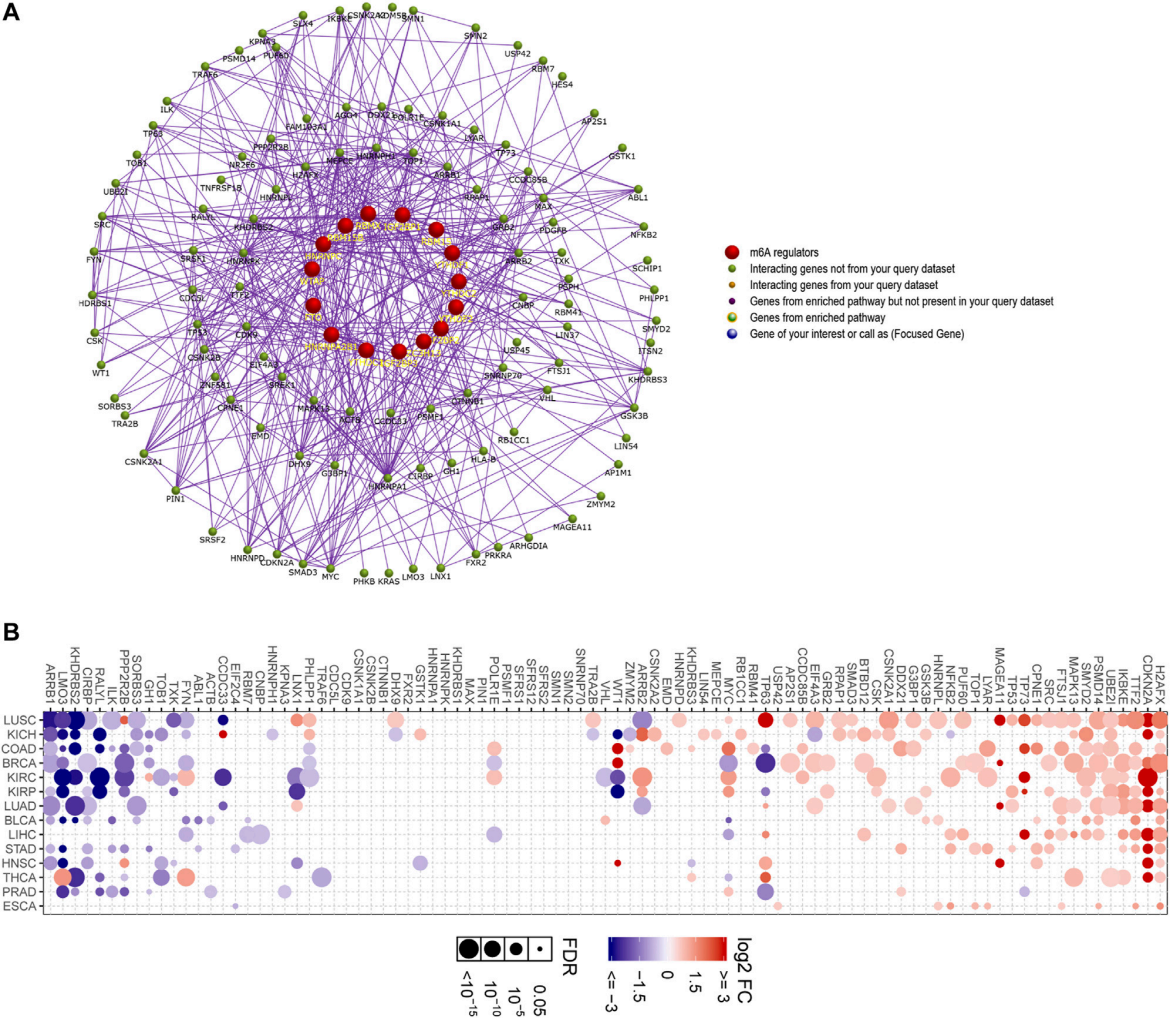
Constructing the interacting regulatory network of m^6^A regulators and their corresponding interacting factors. **(A)** The m^6^A regulators and their corresponding interacting factors were constructed into a complex network by applying FunRich software. **(B)** The expressions of the m^6^A regulators-related genes in diverse cancer types were analyzed using GSCALite database.

Furthermore, a more complex interacting regulatory network including m^6^A regulators and their corresponding interacting factors was constructed by applying FunRich software. This network not only validated that m^6^A regulators interacted with other relevant factors, but also revealed that the erasers, writers, and readers interacted with each other frequently (Figure 9A). In addition, we then attempted to explore the expressions of the m^6^A regulators-related genes in that network using GSCALite database, because these genes might be the downstream targets of the m^6^A regulators or even the modulators of the m^6^A regulators, and their dysregulation should be critical in m^6^A regulation. According to the data, numerous genes (such as H2AFX, CDKN2A, TTF2, IKBKE, and UBE2I) were markedly upregulated in many cancer types, while some genes (such as ARRB1, LMO3, KHDRBS2, CIRBP, and RALYL) were remarkably downregulated in multiple cancer types (Figure 9B). Therefore, these data suggested that the majority of the m^6^A regulators-related genes were dysregulated across many cancer types.

**FIGURE 10:**
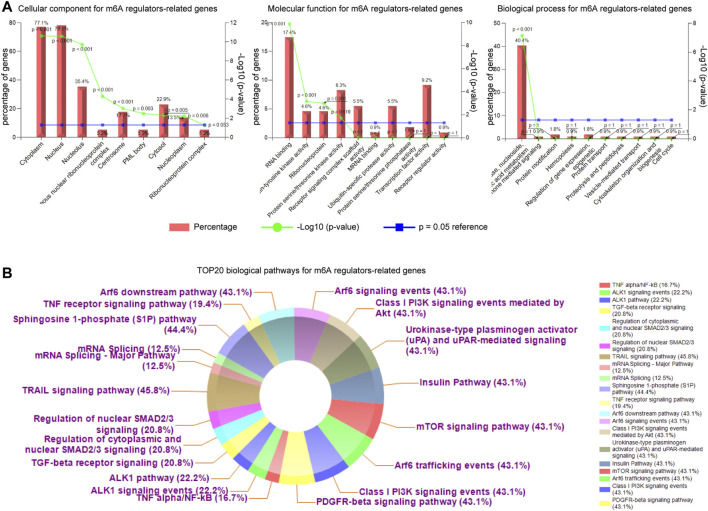
The analyses of gene ontology and biological pathways of the m^6^A regulators-related genes. **(A)** The cellular component (CC), molecular function (MF), and biological process (BP) analyses for the m^6^A regulators-related genes. **(B)** The top 20 biological pathways for m^6^A regulators-related genes (ranked by percentages) were exhibited by doughnut plots using FunRich software.

Next, we aimed to investigate the gene ontology analyses (GO analyses; including three sub-analyses: CC: cellular component; MF: molecular function; BP: biological process) and biological pathways of the m^6^A regulators-related genes. To achieve that, the FunRich software was utilized. The gene ontology analyses revealed that the m^6^A regulators-related genes were significantly associated with cytoplasm (CC), nucleus (CC), RNA binding (MF), regulation of nucleobase, nucleoside, nucleotide and nucleic acid metabolism (BP) (Figure 10A). Moreover, the top 20 biological pathways for m^6^A regulators-related genes (ranked by percentages) were exhibited by doughnut plots and the data showed that these genes were dramatically correlated with many tumorigenesis-relevant pathways, such as TRAIL signaling pathway, S1P pathway, and mTOR signaling (Figure 10B). Therefore, these results indicated that m^6^A regulators as well as their relevant genes were potentially associated with cancers.

## Discussion

Cancers account for the major public health problems, and it leads to the second cause of death, ranking behind cardiovascular diseases, in most countries (Siegel et al., 2019). Therefore, seeking novel approaches for cancer therapy is urgent. Based on deeply understanding the molecular mechanisms, several effectively new methods for treating cancers such as cellular immuno-therapy and PD1/PDL1 antibodies therapy had emerged currently (Sharpe and Pauken, 2018). Moreover, epigenetics including m^6^A methylation, as a popular field of cancer research, might also emerge as a new approach for cancer treatment if its detailed molecular mechanisms in tumorigenesis were deeply unraveled (Zhao et al., 2020). Hence, it is necessary to investigate the effects of m^6^A RNA methylation regulators on multiple cancer types. In the present study, we applied bioinformatics analyses to explore the expression, genetic alterations, prognosis significance, the networks between m^6^A regulators, and potential chemical drugs, miRNAs, or upstream transcriptional factors in multiple cancer types, which deeply uncovered the critical roles and molecular mechanisms of m^6^A regulators in cancers.

Emerging lines of evidence had indicated that the m^6^A regulators served as critical roles in regulating numerous biological processes, diseases, and especially tumor development. For example, m^6^A regulator YTHDF1 was recently identified as a novel prognostic marker and potential target for HCC (Bian et al., 2020). Moreover, m^6^A regulator HNRNPA2B1 was found to function as an oncogenic factor to accelerate esophageal cancer (ESCA) progression, and it might be a promising prognostic biomarker for ESCA (Guo et al., 2020). In addition, the low expression of METTL3, an important m^6^A writer, was found to be correlated with the poor prognosis of triple-negative breast cancer (TNBC), and METTL3 might serve as a novel therapeutic target in TNBC metastasis (Shi et al., 2020). In the present study, our bioinformatics analysis also revealed that YTHDF1, HNRNPA2B1, and METTL3 were highly expressed in colon adenocarcinoma (COAD), lung squamous cell carcinoma (LUSC), and liver hepatocellular carcinoma (LIHC), respectively, and the expression of many other m^6^A regulators such as IGF2BP1, IGF2BP2, and IGF2BP3 was remarkably dysregulated across multiple cancer types.

Deeply understanding cancer hallmarks requires the detailed information of molecular alterations at multiple dimensions such as gene expression, genetic alteration, epigenomics, clinical information, and metabolome. Therefore, the multi-omics analysis approaches were particularly valuable to deeply discover the molecular alterations in pan-cancer. For example, a multi-omics approach was applied to characterize brain metastasis, and the findings revealed that two molecular subtypes showed notably differential prognosis irrespective of brain tumor subtype (Su et al., 2020). Besides, single-cell sequencing was also an important aspect of multi-omics analysis. Recently, several single-cell datasets, including CancerSEA and scLM, were developed to facilitate the mechanism discovery and understanding of complex biosystems such as in cancers (Yuan et al., 2019; Song et al., 2020). In the present study, although lacking single-cell sequencing data, we also applied the multi-omics analysis to uncover the molecular mechanisms of m^6^A regulators in pan-cancer at levels of gene expression, genetic alteration, epigenomics, and clinical information, which might help to facilitate the deep understanding of the modulating mechanisms of m^6^A regulators in pan-cancer.

Up to now, the great majority of the studies focused on researching one or several m^6^A RNA methylation regulators in one or several cancer types (Barbieri et al., 2017; Huang et al., 2019). However, the m^6^A regulators exerted their functions in tumor development and might also collaborate with each other or other factors, and accumulating lines of evidence had indicated that m^6^A regulators might play a dual role as tumor promoters or tumor suppressors in variously different cancer types, implying that the levels or functions of m^6^A RNA methylation were determined by the collaboration of m^6^A regulators in certain conditions (Roundtree et al., 2017; Panneerdoss et al., 2018). Therefore, the comprehensive analyses of all the m^6^A regulators but not several of them across all the cancer types might help supply unique insights into the molecular mechanisms of m^6^A RNA methylation in many cancer types. In the present study, the landscapes of the gene expression, genetic alterations, the prognosis significance, and interacting networks of the 20 m^6^A regulators across dozens of cancer types were revealed by integrative bioinformatics analyses. These results provided new supplementary knowledge about the modulation of the m^6^A regulators’ dysregulation across cancer types and novel insights into the possible molecular mechanisms of the m^6^A regulators’ dysregulation in TCGA cancer types.

The expression alteration of m^6^A regulators in various cancer types might provide novel insight into the molecular mechanisms of tumorigenesis and new therapy approaches (Chen and Wong, 2020). In addition, many aspects such as genetic alternations, epigenetics, and transcriptional factors could contribute to the dysregulation of the m^6^A regulators in cancers (Li et al., 2019). For example, the m^6^A levels were increased through miR-145 targeting YTHDF2, which caused the suppression of cancer cell proliferation in HCC (Yang et al., 2017). Another study demonstrated that SPI1, as a transcriptional factor in hematopoietic cancer cells, could directly suppress the expression of METTL14 (Weng et al., 2018). Therefore, in this study, we not only explored that the methylation and genetic alterations were capable of affecting the expression of the m^6^A regulators across TCGA cancer types, but also investigated the potential upstream miRNAs and transcriptional factors that were able to target these m^6^A regulators. Our results including miRNAs or TF–m^6^A regulators networks provided new supplementary knowledge about the modulation of the m^6^A regulators’ dysregulation across cancer types.

The dysregulation of m^6^A regulators was involved in the procedures of cancer development (Huang et al., 2020). Hence, discovering novel drugs targeting these m^6^A regulators was critical for cancer therapy. For example, a chemical compound, MA2, as an inhibitor of FTO, could effectively suppress the tumor progression of glioblastoma (Cui et al., 2017). Besides, FB23-2 was also capable of inhibiting FTO expression to suppress the proliferation of AML cells (Huang et al., 2019). In the present study, we thereby investigated whether there were some potential chemicals that could increase or decrease the expression of the m^6^A regulators. By analyzing the chemical database, the m^6^A regulators–potential drugs network was constructed and it might provide benefits for potential drugs discovery to target specific m^6^A regulators.

## Conclusion

In summary, our results not only systematically analyze the expression, genetic alterations, oncogenic pathways, and prognosis significance of m^6^A regulators across multiple cancer types, but also constructed the networks between m^6^A regulators and potential chemical drugs, miRNAs, or upstream transcriptional factors. These comprehensive analyses might provide novel understanding of these m^6^A regulators’ roles and shed light on their potential molecular mechanisms in cancers as well as help develop new therapy approaches for cancers.

## Data Availability

The original contributions presented in the study are included in the article/Supplementary Material, further inquiries can be directed to the corresponding author.

## References

[B1] Agarwal V. Bell G. W. Nam J.-W. Bartel D. P. (2015). Predicting Effective microRNA Target Sites in Mammalian mRNAs. Elife 4, e05005. 10.7554/eLife.05005 PMC4532895 26267216

[B2] Barbieri I. Tzelepis K. Pandolfini L. Shi J. Millán-Zambrano G. Robson S. C. (2017). Promoter-bound METTL3 Maintains Myeloid Leukaemia by m6A-dependent Translation Control. Nature 552 (7683), 126–131. 10.1038/nature24678 29186125 PMC6217924

[B3] Bian S. Ni W. Zhu M. Song Q. Zhang J. Ni R. (2020). Identification and Validation of the N6-Methyladenosine RNA Methylation Regulator YTHDF1 as a Novel Prognostic Marker and Potential Target for Hepatocellular Carcinoma. Front. Mol. Biosci. 7, 604766. 10.3389/fmolb.2020.604766 33363211 PMC7758441

[B4] Cerami E. Gao J. Dogrusoz U. Gross B. E. Sumer S. O. Aksoy B. A. (2012). The cBio Cancer Genomics Portal: An Open Platform for Exploring Multidimensional Cancer Genomics Data: Figure 1. Cancer Discov. 2 (5), 401–404. 10.1158/2159-8290.CD-12-0095 22588877 PMC3956037

[B5] Chandrashekar D. S. Bashel B. Balasubramanya S. A. H. Creighton C. J. Ponce-Rodriguez I. Chakravarthi B. V. S. K. (2017). UALCAN: A Portal for Facilitating Tumor Subgroup Gene Expression and Survival Analyses. Neoplasia 19 (8), 649–658. 10.1016/j.neo.2017.05.002 28732212 PMC5516091

[B6] Chen M. Wei L. Law C.-T. Tsang F. H.-C. Shen J. Cheng C. L.-H. (2018). RNA N6-Methyladenosine Methyltransferase-like 3 Promotes Liver Cancer Progression through YTHDF2-dependent Posttranscriptional Silencing of SOCS2. Hepatology 67 (6), 2254–2270. 10.1002/hep.29683 29171881

[B7] Chen M. Wong C.-M. (2020). The Emerging Roles of N6-Methyladenosine (m6A) Deregulation in Liver Carcinogenesis. Mol. Cancer 19 (1), 44. 10.1186/s12943-020-01172-y 32111216 PMC7047367

[B8] Chen X.-Y. Zhang J. Zhu J.-S. (2019). The Role of m6A RNA Methylation in Human Cancer. Mol. Cancer 18 (1), 103. 10.1186/s12943-019-1033-z 31142332 PMC6540575

[B9] Chen Y. Wang X. (2020). miRDB: an Online Database for Prediction of Functional microRNA Targets. Nucleic Acids Res. 48 (D1), D127–D131. 10.1093/nar/gkz757 31504780 PMC6943051

[B10] Cui Q. Shi H. Ye P. Li L. Qu Q. Sun G. (2017). m 6 A RNA Methylation Regulates the Self-Renewal and Tumorigenesis of Glioblastoma Stem Cells. Cel Rep. 18 (11), 2622–2634. 10.1016/j.celrep.2017.02.059 PMC5479356 28297667

[B11] Davis A. P. Grondin C. J. Johnson R. J. Sciaky D. Wiegers J. Wiegers T. C. (2021). Comparative Toxicogenomics Database (CTD): Update 2021. Nucleic Acids Res. 49 (D1), D1138–D1143. 10.1093/nar/gkaa891 33068428 PMC7779006

[B12] De Jesus D. F. Zhang Z. Kahraman S. Brown N. K. Chen M. Hu J. (2019). m6A mRNA Methylation Regulates Human β-cell Biology in Physiological States and in Type 2 Diabetes. Nat. Metab. 1 (8), 765–774. 10.1038/s42255-019-0089-9 31867565 PMC6924515

[B13] Feng C. Song C. Liu Y. Qian F. Gao Y. Ning Z. (2020). KnockTF: a Comprehensive Human Gene Expression Profile Database with Knockdown/knockout of Transcription Factors. Nucleic Acids Res. 48 (D1), D93–D100. 10.1093/nar/gkz881 31598675 PMC6943067

[B14] Guo H. Wang B. Xu K. Nie L. Fu Y. Wang Z. (2020). m6A Reader HNRNPA2B1 Promotes Esophageal Cancer Progression via Up-Regulation of ACLY and ACC1. Front. Oncol. 10, 553045. 10.3389/fonc.2020.553045 33134163 PMC7550530

[B15] He L. Li H. Wu A. Peng Y. Shu G. Yin G. (2019). Functions of N6-Methyladenosine and its Role in Cancer. Mol. Cancer 18 (1), 176. 10.1186/s12943-019-1109-9 31801551 PMC6892141

[B16] He P. C. He C. (2021). m 6 A RNA Methylation: from Mechanisms to Therapeutic Potential. EMBO J. 40 (3), e105977. 10.15252/embj.2020105977 33470439 PMC7849164

[B17] Huang H. Weng H. Chen J. (2020). m6A Modification in Coding and Non-coding RNAs: Roles and Therapeutic Implications in Cancer. Cancer Cell 37 (3), 270–288. 10.1016/j.ccell.2020.02.004 32183948 PMC7141420

[B18] Huang Y. Su R. Sheng Y. Dong L. Dong Z. Xu H. (2019). Small-Molecule Targeting of Oncogenic FTO Demethylase in Acute Myeloid Leukemia. Cancer Cell 35 (4), 677–691. 10.1016/j.ccell.2019.03.006 30991027 PMC6812656

[B19] Li J.-H. Liu S. Zhou H. Qu L.-H. Yang J.-H. (2014). starBase v2.0: Decoding miRNA-ceRNA, miRNA-ncRNA and Protein-RNA Interaction Networks from Large-Scale CLIP-Seq Data. Nucl. Acids Res. 42, D92–D97. 10.1093/nar/gkt1248 24297251 PMC3964941

[B20] Li Y. Xiao J. Bai J. Tian Y. Qu Y. Chen X. (2019). Molecular Characterization and Clinical Relevance of m6A Regulators across 33 Cancer Types. Mol. Cancer 18 (1), 137. 10.1186/s12943-019-1066-3 31521193 PMC6744659

[B21] Liu C.-J. Hu F.-F. Xia M.-X. Han L. Zhang Q. Guo A.-Y. (2018). GSCALite: a Web Server for Gene Set Cancer Analysis. Bioinformatics 34 (21), 3771–3772. 10.1093/bioinformatics/bty411 29790900

[B22] Ma H. Wang X. Cai J. Dai Q. Natchiar S. K. Lv R. (2019). N6-Methyladenosine Methyltransferase ZCCHC4 Mediates Ribosomal RNA Methylation. Nat. Chem. Biol. 15 (1), 88–94. 10.1038/s41589-018-0184-3 30531910 PMC6463480

[B23] Meyer K. D. Jaffrey S. R. (2017). Rethinking m6A Readers, Writers, and Erasers. Annu. Rev. Cel Dev. Biol. 33, 319–342. 10.1146/annurev-cellbio-100616-060758 PMC5963928 28759256

[B24] Meyer K. D. Saletore Y. Zumbo P. Elemento O. Mason C. E. Jaffrey S. R. (2012). Comprehensive Analysis of mRNA Methylation Reveals Enrichment in 3′ UTRs and Near Stop Codons. Cell 149 (7), 1635–1646. 10.1016/j.cell.2012.05.003 22608085 PMC3383396

[B25] Panneerdoss S. Eedunuri V. K. Yadav P. Timilsina S. Rajamanickam S. Viswanadhapalli S. (2018). Cross-talk Among Writers, Readers, and Erasers of M 6 A Regulates Cancer Growth and Progression. Sci. Adv. 4 (10), eaar8263. 10.1126/sciadv.aar8263 30306128 PMC6170038

[B26] Paris J. Morgan M. Campos J. Spencer G. J. Shmakova A. Ivanova I. (2019). Targeting the RNA m6A Reader YTHDF2 Selectively Compromises Cancer Stem Cells in Acute Myeloid Leukemia. Cell Stem Cell 25 (1), 137–148. 10.1016/j.stem.2019.03.021 31031138 PMC6617387

[B27] Pathan M. Keerthikumar S. Ang C.-S. Gangoda L. Quek C. Y. J. Williamson N. A. (2015). FunRich: An Open Access Standalone Functional Enrichment and Interaction Network Analysis Tool. Proteomics 15 (15), 2597–2601. 10.1002/pmic.201400515 25921073

[B28] Roundtree I. A. Evans M. E. Pan T. He C. (2017). Dynamic RNA Modifications in Gene Expression Regulation. Cell 169 (7), 1187–1200. 10.1016/j.cell.2017.05.045 28622506 PMC5657247

[B29] Shannon P. Markiel A. Ozier O. Baliga N. S. Wang J. T. Ramage D. (2003). Cytoscape: a Software Environment for Integrated Models of Biomolecular Interaction Networks. Genome Res. 13 (11), 2498–2504. 10.1101/gr.1239303 14597658 PMC403769

[B30] Sharpe A. H. Pauken K. E. (2018). The Diverse Functions of the PD1 Inhibitory Pathway. Nat. Rev. Immunol. 18 (3), 153–167. 10.1038/nri.2017.108 28990585

[B31] Shi Y. Zheng C. Jin Y. Bao B. Wang D. Hou K. (2020). Reduced Expression of METTL3 Promotes Metastasis of Triple-Negative Breast Cancer by m6A Methylation-Mediated COL3A1 Up-Regulation. Front. Oncol. 10, 1126. 10.3389/fonc.2020.01126 32766145 PMC7381173

[B32] Siegel R. L. Miller K. D. Jemal A. (2019). Cancer Statistics, 2019. CA A. Cancer J. Clin. 69 (1), 7–34. 10.3322/caac.21551 30620402

[B33] Song Q. Su J. Miller L. D. Zhang W. (2021). scLM: Automatic Detection of Consensus Gene Clusters across Multiple Single-Cell Datasets. Genomics, Proteomics & Bioinformatics 19, 330–341. 10.1016/j.gpb.2020.09.002 PMC8602751 33359676

[B34] Su J. Song Q. Qasem S. O’Neill S. Lee J. Furdui C. M. (2020). Multi-Omics Analysis of Brain Metastasis Outcomes Following Craniotomy. Front. Oncol. 10, 615472. 10.3389/fonc.2020.615472 33889540 PMC8056216

[B35] Sun T. Wu R. Ming L. (2019). The Role of m6A RNA Methylation in Cancer. Biomed. Pharmacother. 112, 108613. 10.1016/j.biopha.2019.108613 30784918

[B36] Szklarczyk D. Gable A. L. Lyon D. Junge A. Wyder S. Huerta-Cepas J. (2019). STRING V11: Protein-Protein Association Networks with Increased Coverage, Supporting Functional Discovery in Genome-wide Experimental Datasets. Nucleic Acids Res. 47 (D1), D607–D613. 10.1093/nar/gky1131 30476243 PMC6323986

[B37] Weng H. Huang H. Wu H. Qin X. Zhao B. S. Dong L. (2018). METTL14 Inhibits Hematopoietic Stem/Progenitor Differentiation and Promotes Leukemogenesis via mRNA m6A Modification. Cell Stem Cell 22 (2), 191–205. 10.1016/j.stem.2017.11.016 29290617 PMC5860916

[B38] Yang Z. Li J. Feng G. Gao S. Wang Y. Zhang S. (2017). MicroRNA-145 Modulates N6-Methyladenosine Levels by Targeting the 3′-Untranslated mRNA Region of the N6-Methyladenosine Binding YTH Domain Family 2 Protein. J. Biol. Chem. 292 (9), 3614–3623. 10.1074/jbc.M116.749689 28104805 PMC5339747

[B39] Yuan H. Yan M. Zhang G. Liu W. Deng C. Liao G. (2019). CancerSEA: a Cancer Single-Cell State Atlas. Nucleic Acids Res. 47 (D1), D900–D908. 10.1093/nar/gky939 30329142 PMC6324047

[B40] Zaccara S. Ries R. J. Jaffrey S. R. (2019). Reading, Writing and Erasing mRNA Methylation. Nat. Rev. Mol. Cel Biol 20 (10), 608–624. 10.1038/s41580-019-0168-5 31520073

[B41] Zhang S. Zhao B. S. Zhou A. Lin K. Zheng S. Lu Z. (2017). m 6 A Demethylase ALKBH5 Maintains Tumorigenicity of Glioblastoma Stem-like Cells by Sustaining FOXM1 Expression and Cell Proliferation Program. Cancer Cell 31 (4), 591–606. 10.1016/j.ccell.2017.02.013 28344040 PMC5427719

[B42] Zhao B. S. Roundtree I. A. He C. (2017). Post-transcriptional Gene Regulation by mRNA Modifications. Nat. Rev. Mol. Cel Biol 18 (1), 31–42. 10.1038/nrm.2016.132 PMC5167638 27808276

[B43] Zhao W. Qi X. Liu L. Ma S. Liu J. Wu J. (2020). Epigenetic Regulation of m6A Modifications in Human Cancer. Mol. Ther. - Nucleic Acids 19, 405–412. 10.1016/j.omtn.2019.11.022 31887551 PMC6938965

