# Supplementary material for: Comprehensive Analyses of the Expression, Genetic Alteration, Prognosis Significance, and Interaction Networks of m6A Regulators Across Human Cancers
Source: Front Genet. 2021 Dec 23;12:771853. doi: 10.3389/fgene.2021.771853 (PMC8733627; doi:10.3389/fgene.2021.771853)
Supplement: Supplementary file 1 [file DataSheet1.docx]

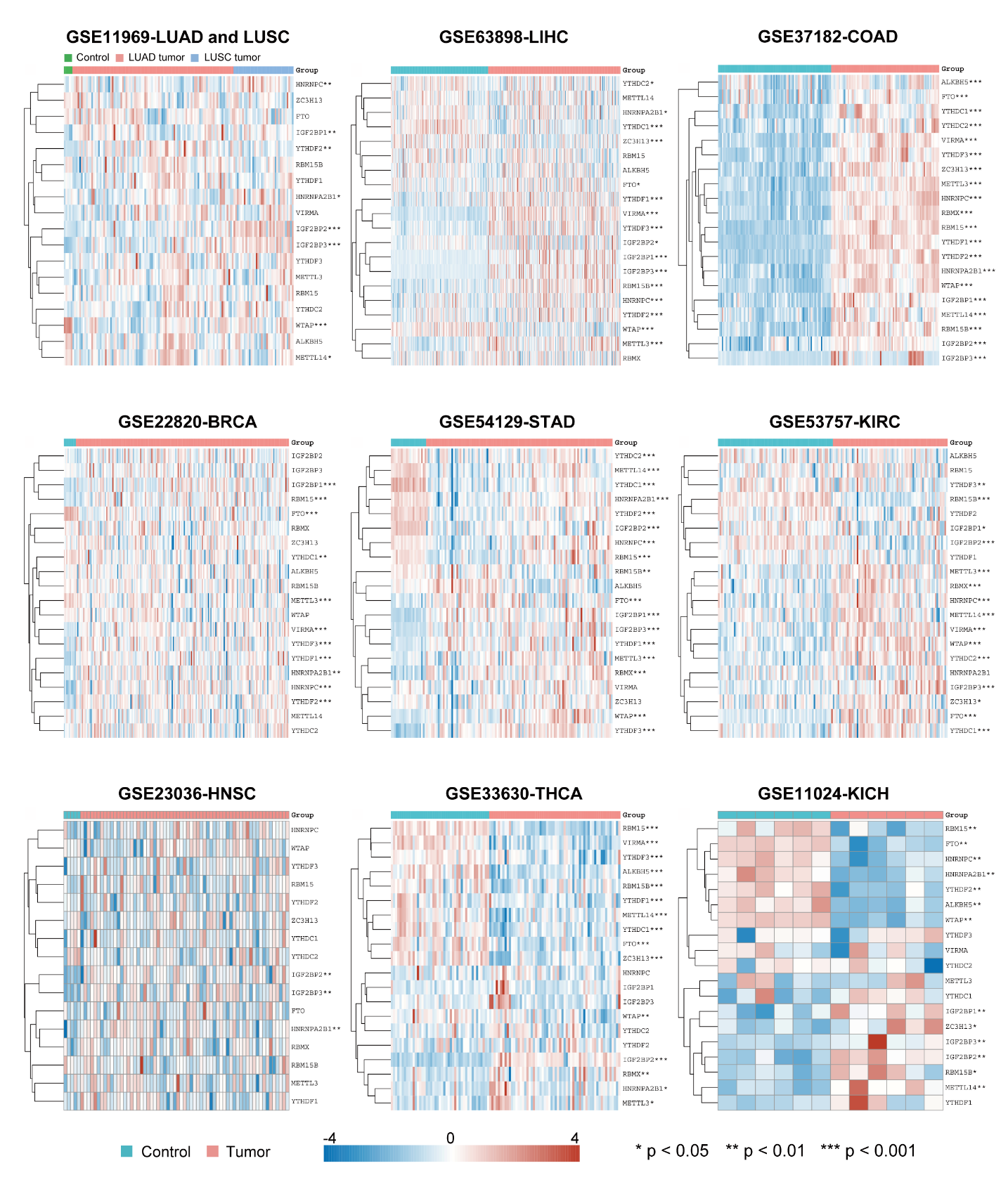
Supplementary Material

**Supplementary Figure 1.** The heat-maps of m6A regulators from GEO datasets.


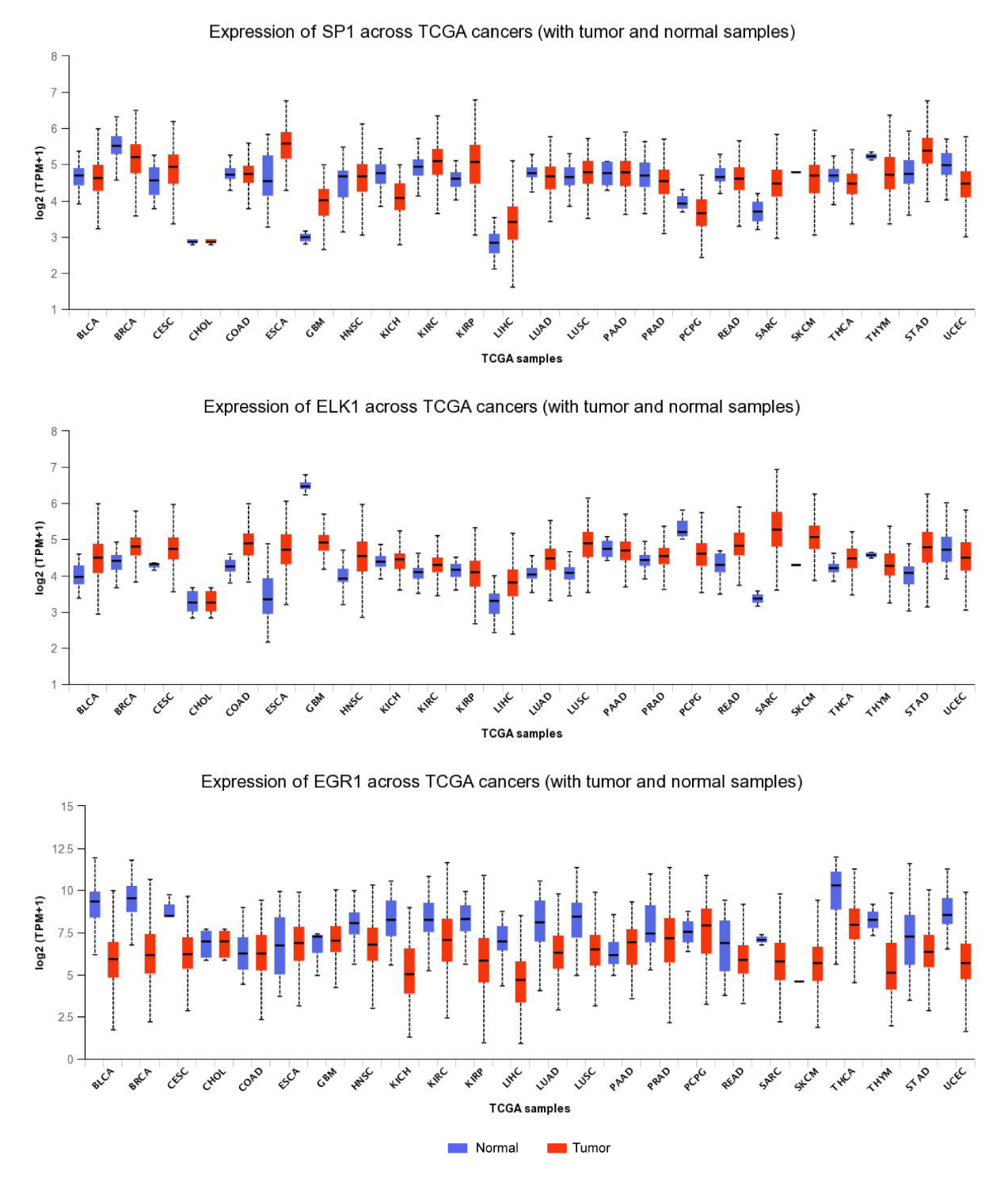


**Supplementary Figure 2.** The expressions of SP1, ELK1 and EGR1 were analyzed across TCGA cancer types using UALCAN database.

**Supplementary Table 1.** The chemicals were able to increase or decrease the expression of the m^6^A regulators.

| Chemical Name Chemical ID Gene Symbol Interaction Actions |
| --- |
| \|  \|  \|  \|  \| \| --- \| --- \| --- \| --- \| \| abrine \| C496492 \| ALKBH5 \| decreases \| \| aflatoxin B2 \| C029753 \| ALKBH5 \| increases \| \| cobaltous chloride \| C018021 \| ALKBH5 \| increases \| \| Formaldehyde \| D005557 \| ALKBH5 \| decreases \| \| (+)-JQ1 compound \| C561695 \| ALKBH5 \| increases \| \| Methyl Methanesulfonate \| D008741 \| ALKBH5 \| decreases \| \| MLN7243 \| C000622638 \| ALKBH5 \| increases \| \| Sunitinib \| D000077210 \| ALKBH5 \| increases \| \| Urethane \| D014520 \| ALKBH5 \| increases \| \| 3-(2-hydroxy-4-(2-methylnonan-2-yl)phenyl)cyclohexan-1-ol \| C572491 \| ZC3H13 \| decreases \| \| 4-(5-benzo(1,3)dioxol-5-yl-4-pyridin-2-yl-1H-imidazol-2-yl)benzamide \| C459179 \| ZC3H13 \| decreases \| \| abrine \| C496492 \| ZC3H13 \| decreases \| \| Acetaminophen \| D000082 \| ZC3H13 \| decreases \| \| Cisplatin \| D002945 \| ZC3H13 \| decreases \| \| dorsomorphin \| C516138 \| ZC3H13 \| decreases \| \| Formaldehyde \| D005557 \| ZC3H13 \| decreases \| \| Indomethacin \| D007213 \| ZC3H13 \| decreases \| \| nickel sulfate \| C029938 \| ZC3H13 \| decreases \| \| pentanal \| C046012 \| ZC3H13 \| decreases \| \| Phenylmercuric Acetate \| D010662 \| ZC3H13 \| decreases \| \| Plant Extracts \| D010936 \| ZC3H13 \| increases \| \| Valproic Acid \| D014635 \| ZC3H13 \| decreases \| \| abrine \| C496492 \| ALKBH5 \| decreases \| \| aflatoxin B2 \| C029753 \| ALKBH5 \| increases \| \| cobaltous chloride \| C018021 \| ALKBH5 \| increases \| \| Formaldehyde \| D005557 \| ALKBH5 \| decreases \| \| (+)-JQ1 compound \| C561695 \| ALKBH5 \| increases \| \| Methyl Methanesulfonate \| D008741 \| ALKBH5 \| decreases \| \| MLN7243 \| C000622638 \| ALKBH5 \| increases \| \| Sunitinib \| D000077210 \| ALKBH5 \| increases \| \| Urethane \| D014520 \| ALKBH5 \| increases \| \| 1-Methyl-3-isobutylxanthine \| D015056 \| FTO \| increases \| \| 7,8-Dihydro-7,8-dihydroxybenzo(a)pyrene 9,10-oxide \| D015123 \| FTO \| decreases \| \| Antirheumatic Agents \| D018501 \| FTO \| increases \| \| Benzo(a)pyrene \| D001564 \| FTO \| decreases \| \| bisphenol F \| C000611646 \| FTO \| increases \| \| butyraldehyde \| C018475 \| FTO \| decreases \| \| Cisplatin \| D002945 \| FTO \| decreases \| \| Dexamethasone \| D003907 \| FTO \| increases \| \| dicrotophos \| C000944 \| FTO \| increases \| \| Formaldehyde \| D005557 \| FTO \| decreases \| \| Indomethacin \| D007213 \| FTO \| increases \| \| Irinotecan \| D000077146 \| FTO \| decreases \| \| Lead \| D007854 \| FTO \| decreases \| \| pentanal \| C046012 \| FTO \| decreases \| \| Tobacco Smoke Pollution \| D014028 \| FTO \| increases \| \| Valproic Acid \| D014635 \| FTO \| increases \| \| Vanadates \| D014638 \| FTO \| increases \| \| 4-(5-benzo(1,3)dioxol-5-yl-4-pyridin-2-yl-1H-imidazol-2-yl)benzamide \| C459179 \| HNRNPA2B1 \| increases \| \| (4-amino-1,4-dihydro-3-(2-pyridyl)-5-thioxo-1,2,4-triazole)copper(II) \| C517041 \| HNRNPA2B1 \| increases \| \| 4-phenylbutyric acid \| C075773 \| HNRNPA2B1 \| decreases \| \| 7,8-Dihydro-7,8-dihydroxybenzo(a)pyrene 9,10-oxide \| D015123 \| HNRNPA2B1 \| increases \| \| Acetaminophen \| D000082 \| HNRNPA2B1 \| increases \| \| afimoxifene \| C016601 \| HNRNPA2B1 \| decreases \| \| Alitretinoin \| D000077556 \| HNRNPA2B1 \| decreases \| \| Arsenic Trioxide \| D000077237 \| HNRNPA2B1 \| decreases \| \| Benzo(a)pyrene \| D001564 \| HNRNPA2B1 \| increases \| \| bisphenol A \| C006780 \| HNRNPA2B1 \| decreases \| \| butyraldehyde \| C018475 \| HNRNPA2B1 \| decreases \| \| Cisplatin \| D002945 \| HNRNPA2B1 \| decreases \| \| Coal Tar \| D003033 \| HNRNPA2B1 \| increases \| \| cobaltous chloride \| C018021 \| HNRNPA2B1 \| decreases \| \| Copper \| D003300 \| HNRNPA2B1 \| increases \| \| Copper \| D003300 \| HNRNPA2B1 \| decreases \| \| Cyclosporine \| D016572 \| HNRNPA2B1 \| decreases \| \| Decitabine \| D000077209 \| HNRNPA2B1 \| decreases \| \| diallyl trisulfide \| C042577 \| HNRNPA2B1 \| decreases \| \| dibenzo(a,l)pyrene \| C041517 \| HNRNPA2B1 \| increases \| \| dicrotophos \| C000944 \| HNRNPA2B1 \| decreases \| \| Disulfiram \| D004221 \| HNRNPA2B1 \| increases \| \| dorsomorphin \| C516138 \| HNRNPA2B1 \| increases \| \| erucylphospho-N,N,N-trimethylpropylammonium \| C472787 \| HNRNPA2B1 \| decreases \| \| Estradiol \| D004958 \| HNRNPA2B1 \| increases \| \| Ethanol \| D000431 \| HNRNPA2B1 \| increases \| \| eurycomanone \| C506425 \| HNRNPA2B1 \| decreases \| \| Fluorouracil \| D005472 \| HNRNPA2B1 \| decreases \| \| Formaldehyde \| D005557 \| HNRNPA2B1 \| decreases \| \| Fulvestrant \| D000077267 \| HNRNPA2B1 \| decreases \| \| Genistein \| D019833 \| HNRNPA2B1 \| increases \| \| Graphite \| D006108 \| HNRNPA2B1 \| increases \| \| ICG 001 \| C492448 \| HNRNPA2B1 \| decreases \| \| (+)-JQ1 compound \| C561695 \| HNRNPA2B1 \| decreases \| \| K 7174 \| C410337 \| HNRNPA2B1 \| decreases \| \| methylmercuric chloride \| C004925 \| HNRNPA2B1 \| increases \| \| Methyl Methanesulfonate \| D008741 \| HNRNPA2B1 \| decreases \| \| MK2i peptide \| C540575 \| HNRNPA2B1 \| decreases \| \| Nanotubes, Carbon \| D037742 \| HNRNPA2B1 \| increases \| \| NSC 689534 \| C558013 \| HNRNPA2B1 \| decreases \| \| Paclitaxel \| D017239 \| HNRNPA2B1 \| decreases \| \| p-Chloromercuribenzoic Acid \| D020245 \| HNRNPA2B1 \| increases \| \| pentanal \| C046012 \| HNRNPA2B1 \| decreases \| \| Plant Extracts \| D010936 \| HNRNPA2B1 \| increases \| \| Quercetin \| D011794 \| HNRNPA2B1 \| decreases \| \| Quercetin \| D011794 \| HNRNPA2B1 \| increases \| \| Raloxifene Hydrochloride \| D020849 \| HNRNPA2B1 \| decreases \| \| Sunitinib \| D000077210 \| HNRNPA2B1 \| decreases \| \| Thapsigargin \| D019284 \| HNRNPA2B1 \| decreases \| \| Tretinoin \| D014212 \| HNRNPA2B1 \| increases \| \| trichostatin A \| C012589 \| HNRNPA2B1 \| decreases \| \| tris(1,3-dichloro-2-propyl)phosphate \| C016805 \| HNRNPA2B1 \| decreases \| \| Tunicamycin \| D014415 \| HNRNPA2B1 \| decreases \| \| Urethane \| D014520 \| HNRNPA2B1 \| decreases \| \| Valproic Acid \| D014635 \| HNRNPA2B1 \| decreases \| \| vanadyl sulfate \| C034028 \| HNRNPA2B1 \| decreases \| \| Vehicle Emissions \| D001335 \| HNRNPA2B1 \| increases \| \| Vincristine \| D014750 \| HNRNPA2B1 \| decreases \| \| 4-(5-benzo(1,3)dioxol-5-yl-4-pyridin-2-yl-1H-imidazol-2-yl)benzamide \| C459179 \| HNRNPC \| decreases \| \| 4-phenylbutyric acid \| C075773 \| HNRNPC \| decreases \| \| abrine \| C496492 \| HNRNPC \| increases \| \| Acetaminophen \| D000082 \| HNRNPC \| decreases \| \| arsenic disulfide \| C058317 \| HNRNPC \| increases \| \| Asbestos, Crocidolite \| D017638 \| HNRNPC \| increases \| \| Benzo(a)pyrene \| D001564 \| HNRNPC \| decreases \| \| bisphenol A \| C006780 \| HNRNPC \| decreases \| \| bromovanin \| C515564 \| HNRNPC \| increases \| \| C646 compound \| C584509 \| HNRNPC \| decreases \| \| CD 437 \| C099555 \| HNRNPC \| decreases \| \| Cisplatin \| D002945 \| HNRNPC \| decreases \| \| cobaltous chloride \| C018021 \| HNRNPC \| decreases \| \| Copper \| D003300 \| HNRNPC \| decreases \| \| Cyclosporine \| D016572 \| HNRNPC \| decreases \| \| dorsomorphin \| C516138 \| HNRNPC \| decreases \| \| Doxorubicin \| D004317 \| HNRNPC \| decreases \| \| Ethyl Methanesulfonate \| D005020 \| HNRNPC \| decreases \| \| Fluorouracil \| D005472 \| HNRNPC \| increases \| \| Formaldehyde \| D005557 \| HNRNPC \| decreases \| \| Genistein \| D019833 \| HNRNPC \| decreases \| \| Genistein \| D019833 \| HNRNPC \| increases \| \| K 7174 \| C410337 \| HNRNPC \| decreases \| \| Methotrexate \| D008727 \| HNRNPC \| decreases \| \| methylmercuric chloride \| C004925 \| HNRNPC \| decreases \| \| Methyl Methanesulfonate \| D008741 \| HNRNPC \| decreases \| \| Metribolone \| D015741 \| HNRNPC \| decreases \| \| NSC 689534 \| C558013 \| HNRNPC \| decreases \| \| Paraoxon \| D010261 \| HNRNPC \| increases \| \| perfluorooctane sulfonic acid \| C076994 \| HNRNPC \| decreases \| \| Plant Extracts \| D010936 \| HNRNPC \| decreases \| \| Plant Extracts \| D010936 \| HNRNPC \| increases \| \| PP242 \| C572919 \| HNRNPC \| increases \| \| Soot \| D053260 \| HNRNPC \| decreases \| \| Tobacco Smoke Pollution \| D014028 \| HNRNPC \| increases \| \| Tretinoin \| D014212 \| HNRNPC \| decreases \| \| trichostatin A \| C012589 \| HNRNPC \| decreases \| \| Uranium Compounds \| D017974 \| HNRNPC \| decreases \| \| Valproic Acid \| D014635 \| HNRNPC \| decreases \| \| yessotoxin \| C066632 \| HNRNPC \| decreases \| \| 1-Methyl-3-isobutylxanthine \| D015056 \| IGF2BP1 \| decreases \| \| 4-(5-benzo(1,3)dioxol-5-yl-4-pyridin-2-yl-1H-imidazol-2-yl)benzamide \| C459179 \| IGF2BP1 \| increases \| \| abrine \| C496492 \| IGF2BP1 \| increases \| \| Acetaminophen \| D000082 \| IGF2BP1 \| decreases \| \| bis(4-hydroxyphenyl)sulfone \| C543008 \| IGF2BP1 \| decreases \| \| bisphenol F \| C000611646 \| IGF2BP1 \| decreases \| \| butyraldehyde \| C018475 \| IGF2BP1 \| decreases \| \| Cisplatin \| D002945 \| IGF2BP1 \| decreases \| \| cobaltous chloride \| C018021 \| IGF2BP1 \| decreases \| \| Copper Sulfate \| D019327 \| IGF2BP1 \| increases \| \| Dexamethasone \| D003907 \| IGF2BP1 \| decreases \| \| Diazinon \| D003976 \| IGF2BP1 \| increases \| \| dicrotophos \| C000944 \| IGF2BP1 \| increases \| \| dorsomorphin \| C516138 \| IGF2BP1 \| increases \| \| entinostat \| C118739 \| IGF2BP1 \| increases \| \| Estradiol \| D004958 \| IGF2BP1 \| increases \| \| Indomethacin \| D007213 \| IGF2BP1 \| decreases \| \| jinfukang \| C544151 \| IGF2BP1 \| decreases \| \| K 7174 \| C410337 \| IGF2BP1 \| decreases \| \| Magnetite Nanoparticles \| D058185 \| IGF2BP1 \| decreases \| \| methylmercuric chloride \| C004925 \| IGF2BP1 \| increases \| \| nickel sulfate \| C029938 \| IGF2BP1 \| decreases \| \| potassium chromate(VI) \| C027373 \| IGF2BP1 \| increases \| \| sodium arsenite \| C017947 \| IGF2BP1 \| decreases \| \| sodium arsenite \| C017947 \| IGF2BP1 \| increases \| \| Succimer \| D004113 \| IGF2BP1 \| decreases \| \| tris(1,3-dichloro-2-propyl)phosphate \| C016805 \| IGF2BP1 \| decreases \| \| Urethane \| D014520 \| IGF2BP1 \| decreases \| \| Valproic Acid \| D014635 \| IGF2BP1 \| decreases \| \| 4-(5-benzo(1,3)dioxol-5-yl-4-pyridin-2-yl-1H-imidazol-2-yl)benzamide \| C459179 \| IGF2BP2 \| increases \| \| 7,8-Dihydro-7,8-dihydroxybenzo(a)pyrene 9,10-oxide \| D015123 \| IGF2BP2 \| decreases \| \| 8-Bromo Cyclic Adenosine Monophosphate \| D015124 \| IGF2BP2 \| increases \| \| abrine \| C496492 \| IGF2BP2 \| increases \| \| Arsenic Trioxide \| D000077237 \| IGF2BP2 \| decreases \| \| Asbestos, Serpentine \| D017632 \| IGF2BP2 \| increases \| \| C646 compound \| C584509 \| IGF2BP2 \| decreases \| \| Cadmium Chloride \| D019256 \| IGF2BP2 \| increases \| \| chloropicrin \| C100187 \| IGF2BP2 \| increases \| \| Cisplatin \| D002945 \| IGF2BP2 \| decreases \| \| cobaltous chloride \| C018021 \| IGF2BP2 \| decreases \| \| Copper Sulfate \| D019327 \| IGF2BP2 \| increases \| \| Estradiol \| D004958 \| IGF2BP2 \| increases \| \| Hydralazine \| D006830 \| IGF2BP2 \| increases \| \| Methotrexate \| D008727 \| IGF2BP2 \| increases \| \| Particulate Matter \| D052638 \| IGF2BP2 \| decreases \| \| pirinixic acid \| C006253 \| IGF2BP2 \| decreases \| \| Sodium Selenite \| D018038 \| IGF2BP2 \| decreases \| \| tris(1,3-dichloro-2-propyl)phosphate \| C016805 \| IGF2BP2 \| increases \| \| Valproic Acid \| D014635 \| IGF2BP2 \| increases \| \| Vitamin E \| D014810 \| IGF2BP2 \| decreases \| \| 1-Methyl-3-isobutylxanthine \| D015056 \| IGF2BP3 \| decreases \| \| 4-(5-benzo(1,3)dioxol-5-yl-4-pyridin-2-yl-1H-imidazol-2-yl)benzamide \| C459179 \| IGF2BP3 \| decreases \| \| 7,8-Dihydro-7,8-dihydroxybenzo(a)pyrene 9,10-oxide \| D015123 \| IGF2BP3 \| decreases \| \| Aflatoxin B1 \| D016604 \| IGF2BP3 \| increases \| \| Asbestos \| D001194 \| IGF2BP3 \| decreases \| \| Benzo(a)pyrene \| D001564 \| IGF2BP3 \| decreases \| \| bis(4-hydroxyphenyl)sulfone \| C543008 \| IGF2BP3 \| decreases \| \| bisphenol A \| C006780 \| IGF2BP3 \| decreases \| \| bisphenol F \| C000611646 \| IGF2BP3 \| decreases \| \| C646 compound \| C584509 \| IGF2BP3 \| decreases \| \| cobaltous chloride \| C018021 \| IGF2BP3 \| decreases \| \| Cyclosporine \| D016572 \| IGF2BP3 \| decreases \| \| Cyclosporine \| D016572 \| IGF2BP3 \| increases \| \| Dexamethasone \| D003907 \| IGF2BP3 \| decreases \| \| dorsomorphin \| C516138 \| IGF2BP3 \| decreases \| \| Doxorubicin \| D004317 \| IGF2BP3 \| decreases \| \| epigallocatechin gallate \| C045651 \| IGF2BP3 \| decreases \| \| fatostatin \| C545733 \| IGF2BP3 \| decreases \| \| Formaldehyde \| D005557 \| IGF2BP3 \| decreases \| \| ICG 001 \| C492448 \| IGF2BP3 \| decreases \| \| Indomethacin \| D007213 \| IGF2BP3 \| decreases \| \| Lactic Acid \| D019344 \| IGF2BP3 \| decreases \| \| Methyl Methanesulfonate \| D008741 \| IGF2BP3 \| decreases \| \| pirinixic acid \| C006253 \| IGF2BP3 \| increases \| \| potassium chromate(VI) \| C027373 \| IGF2BP3 \| decreases \| \| Quercetin \| D011794 \| IGF2BP3 \| decreases \| \| sodium arsenite \| C017947 \| IGF2BP3 \| increases \| \| tetrahydropalmatine \| C014215 \| IGF2BP3 \| decreases \| \| Thimerosal \| D013849 \| IGF2BP3 \| decreases \| \| Tobacco Smoke Pollution \| D014028 \| IGF2BP3 \| decreases \| \| torcetrapib \| C483909 \| IGF2BP3 \| increases \| \| trichostatin A \| C012589 \| IGF2BP3 \| decreases \| \| Troglitazone \| D000077288 \| IGF2BP3 \| increases \| \| Urethane \| D014520 \| IGF2BP3 \| decreases \| \| Valproic Acid \| D014635 \| IGF2BP3 \| decreases \| \| Vorinostat \| D000077337 \| IGF2BP3 \| decreases \| \| Acetaminophen \| D000082 \| METTL3 \| decreases \| \| Arsenic Trioxide \| D000077237 \| METTL3 \| decreases \| \| Benzo(a)pyrene \| D001564 \| METTL3 \| increases \| \| bisphenol A \| C006780 \| METTL3 \| decreases \| \| Copper Sulfate \| D019327 \| METTL3 \| decreases \| \| Cyclosporine \| D016572 \| METTL3 \| decreases \| \| Demecolcine \| D003703 \| METTL3 \| increases \| \| Doxorubicin \| D004317 \| METTL3 \| decreases \| \| Hydralazine \| D006830 \| METTL3 \| increases \| \| ICG 001 \| C492448 \| METTL3 \| decreases \| \| jinfukang \| C544151 \| METTL3 \| increases \| \| Leflunomide \| D000077339 \| METTL3 \| decreases \| \| Methyl Methanesulfonate \| D008741 \| METTL3 \| decreases \| \| Plant Oils \| D010938 \| METTL3 \| decreases \| \| Sunitinib \| D000077210 \| METTL3 \| increases \| \| Urethane \| D014520 \| METTL3 \| decreases \| \| Valproic Acid \| D014635 \| METTL3 \| increases \| \| Valproic Acid \| D014635 \| METTL3 \| decreases \| \| Vincristine \| D014750 \| METTL3 \| decreases \| \| 7,8-Dihydro-7,8-dihydroxybenzo(a)pyrene 9,10-oxide \| D015123 \| METTL14 \| decreases \| \| Copper Sulfate \| D019327 \| METTL14 \| increases \| \| cupric oxide \| C030973 \| METTL14 \| increases \| \| dicrotophos \| C000944 \| METTL14 \| decreases \| \| Ethyl Methanesulfonate \| D005020 \| METTL14 \| increases \| \| Methyl Methanesulfonate \| D008741 \| METTL14 \| increases \| \| Plant Extracts \| D010936 \| METTL14 \| increases \| \| Urethane \| D014520 \| METTL14 \| increases \| \| Valproic Acid \| D014635 \| METTL14 \| decreases \| \| 2,3-bis(3'-hydroxybenzyl)butyrolactone \| C029497 \| RBM15 \| increases \| \| 7,8-Dihydro-7,8-dihydroxybenzo(a)pyrene 9,10-oxide \| D015123 \| RBM15 \| decreases \| \| Acetaminophen \| D000082 \| RBM15 \| increases \| \| cadmium sulfate \| C037123 \| RBM15 \| decreases \| \| cobaltous chloride \| C018021 \| RBM15 \| increases \| \| Coumestrol \| D003375 \| RBM15 \| increases \| \| Estradiol \| D004958 \| RBM15 \| increases \| \| Formaldehyde \| D005557 \| RBM15 \| decreases \| \| GSK-J4 \| C000593030 \| RBM15 \| increases \| \| Hydrogen Peroxide \| D006861 \| RBM15 \| decreases \| \| ICG 001 \| C492448 \| RBM15 \| decreases \| \| Indomethacin \| D007213 \| RBM15 \| decreases \| \| K 7174 \| C410337 \| RBM15 \| increases \| \| Lactic Acid \| D019344 \| RBM15 \| increases \| \| Nickel \| D009532 \| RBM15 \| increases \| \| PCI 5002 \| C568608 \| RBM15 \| increases \| \| pentabromodiphenyl ether \| C086401 \| RBM15 \| increases \| \| Plant Extracts \| D010936 \| RBM15 \| increases \| \| Resveratrol \| D000077185 \| RBM15 \| increases \| \| Sunitinib \| D000077210 \| RBM15 \| decreases \| \| Tobacco Smoke Pollution \| D014028 \| RBM15 \| increases \| \| Urethane \| D014520 \| RBM15 \| increases \| \| Valproic Acid \| D014635 \| RBM15 \| decreases \| \| Vincristine \| D014750 \| RBM15 \| decreases \| \| butyraldehyde \| C018475 \| RBM15B \| decreases \| \| cobaltous chloride \| C018021 \| RBM15B \| decreases \| \| Copper \| D003300 \| RBM15B \| decreases \| \| Cyclosporine \| D016572 \| RBM15B \| decreases \| \| Diazinon \| D003976 \| RBM15B \| increases \| \| epigallocatechin gallate \| C045651 \| RBM15B \| increases \| \| GSK-J4 \| C000593030 \| RBM15B \| decreases \| \| Methyl Methanesulfonate \| D008741 \| RBM15B \| decreases \| \| NSC 689534 \| C558013 \| RBM15B \| decreases \| \| pentanal \| C046012 \| RBM15B \| decreases \| \| potassium chromate(VI) \| C027373 \| RBM15B \| increases \| \| quercitrin \| C012526 \| RBM15B \| increases \| \| sodium arsenite \| C017947 \| RBM15B \| increases \| \| Sodium Selenite \| D018038 \| RBM15B \| increases \| \| tris(1,3-dichloro-2-propyl)phosphate \| C016805 \| RBM15B \| decreases \| \| Urethane \| D014520 \| RBM15B \| decreases \| \| Valproic Acid \| D014635 \| RBM15B \| decreases \| \| 4-(5-benzo(1,3)dioxol-5-yl-4-pyridin-2-yl-1H-imidazol-2-yl)benzamide \| C459179 \| RBMX \| decreases \| \| 7,8-Dihydro-7,8-dihydroxybenzo(a)pyrene 9,10-oxide \| D015123 \| RBMX \| decreases \| \| Acetaminophen \| D000082 \| RBMX \| decreases \| \| Aldehydes \| D000447 \| RBMX \| decreases \| \| arsenic disulfide \| C058317 \| RBMX \| decreases \| \| Arsenic Trioxide \| D000077237 \| RBMX \| decreases \| \| Arsenic Trioxide \| D000077237 \| RBMX \| increases \| \| bisphenol A \| C006780 \| RBMX \| decreases \| \| butyraldehyde \| C018475 \| RBMX \| decreases \| \| Caffeine \| D002110 \| RBMX \| decreases \| \| CD 437 \| C099555 \| RBMX \| decreases \| \| Cisplatin \| D002945 \| RBMX \| decreases \| \| Coumestrol \| D003375 \| RBMX \| increases \| \| dorsomorphin \| C516138 \| RBMX \| decreases \| \| epigallocatechin gallate \| C045651 \| RBMX \| decreases \| \| nickel acetate \| C119536 \| RBMX \| increases \| \| nickel acetate \| C119536 \| RBMX \| decreases \| \| pentanal \| C046012 \| RBMX \| decreases \| \| Phenylmercuric Acetate \| D010662 \| RBMX \| decreases \| \| PP242 \| C572919 \| RBMX \| increases \| \| propionaldehyde \| C005556 \| RBMX \| decreases \| \| Tobacco Smoke Pollution \| D014028 \| RBMX \| decreases \| \| tris(1,3-dichloro-2-propyl)phosphate \| C016805 \| RBMX \| decreases \| \| Valproic Acid \| D014635 \| RBMX \| decreases \| \| yessotoxin \| C066632 \| RBMX \| decreases \| \| 1-nitropyrene \| C032668 \| VIRMA \| increases \| \| 4-(5-benzo(1,3)dioxol-5-yl-4-pyridin-2-yl-1H-imidazol-2-yl)benzamide \| C459179 \| VIRMA \| increases \| \| 4-(5-benzo(1,3)dioxol-5-yl-4-pyridin-2-yl-1H-imidazol-2-yl)benzamide \| C459179 \| VIRMA \| decreases \| \| 7,8-Dihydro-7,8-dihydroxybenzo(a)pyrene 9,10-oxide \| D015123 \| VIRMA \| increases \| \| dorsomorphin \| C516138 \| VIRMA \| increases \| \| dorsomorphin \| C516138 \| VIRMA \| decreases \| \| jinfukang \| C544151 \| VIRMA \| decreases \| \| Mercuric Chloride \| D008627 \| VIRMA \| increases \| \| methylmercuric chloride \| C004925 \| VIRMA \| decreases \| \| Plant Extracts \| D010936 \| VIRMA \| increases \| \| trichostatin A \| C012589 \| VIRMA \| decreases \| \| Valproic Acid \| D014635 \| VIRMA \| decreases \| \| 2-amino-1-methyl-6-phenylimidazo(4,5-b)pyridine \| C049584 \| WTAP \| decreases \| \| 7,8-Dihydro-7,8-dihydroxybenzo(a)pyrene 9,10-oxide \| D015123 \| WTAP \| decreases \| \| abrine \| C496492 \| WTAP \| increases \| \| Amiodarone \| D000638 \| WTAP \| increases \| \| Antirheumatic Agents \| D018501 \| WTAP \| decreases \| \| Benzo(a)pyrene \| D001564 \| WTAP \| increases \| \| Cadmium Chloride \| D019256 \| WTAP \| increases \| \| CC-8490 \| C495817 \| WTAP \| increases \| \| Cyclosporine \| D016572 \| WTAP \| increases \| \| Fluorouracil \| D005472 \| WTAP \| decreases \| \| Formaldehyde \| D005557 \| WTAP \| increases \| \| Hydrogen Peroxide \| D006861 \| WTAP \| increases \| \| K 7174 \| C410337 \| WTAP \| increases \| \| Methyl Methanesulfonate \| D008741 \| WTAP \| decreases \| \| nickel sulfate \| C029938 \| WTAP \| decreases \| \| sodium arsenite \| C017947 \| WTAP \| decreases \| \| Sodium Selenite \| D018038 \| WTAP \| increases \| \| Sunitinib \| D000077210 \| WTAP \| increases \| \| Tamoxifen \| D013629 \| WTAP \| increases \| \| Theophylline \| D013806 \| WTAP \| increases \| \| Tobacco Smoke Pollution \| D014028 \| WTAP \| increases \| \| Tretinoin \| D014212 \| WTAP \| increases \| \| Valproic Acid \| D014635 \| WTAP \| decreases \| \| Zoledronic Acid \| D000077211 \| WTAP \| increases \| \| 2-amino-1-methyl-6-phenylimidazo(4,5-b)pyridine \| C049584 \| YTHDC1 \| decreases \| \| (4-amino-1,4-dihydro-3-(2-pyridyl)-5-thioxo-1,2,4-triazole)copper(II) \| C517041 \| YTHDC1 \| increases \| \| Antirheumatic Agents \| D018501 \| YTHDC1 \| increases \| \| Arsenic Trioxide \| D000077237 \| YTHDC1 \| increases \| \| Asbestos, Crocidolite \| D017638 \| YTHDC1 \| increases \| \| Cisplatin \| D002945 \| YTHDC1 \| decreases \| \| Copper Sulfate \| D019327 \| YTHDC1 \| increases \| \| epigallocatechin gallate \| C045651 \| YTHDC1 \| increases \| \| GSK-J4 \| C000593030 \| YTHDC1 \| increases \| \| jinfukang \| C544151 \| YTHDC1 \| decreases \| \| K 7174 \| C410337 \| YTHDC1 \| increases \| \| potassium chromate(VI) \| C027373 \| YTHDC1 \| increases \| \| Rotenone \| D012402 \| YTHDC1 \| decreases \| \| Silver \| D012834 \| YTHDC1 \| increases \| \| Sodium Selenite \| D018038 \| YTHDC1 \| increases \| \| Theophylline \| D013806 \| YTHDC1 \| increases \| \| Tobacco Smoke Pollution \| D014028 \| YTHDC1 \| increases \| \| Tretinoin \| D014212 \| YTHDC1 \| decreases \| \| Valproic Acid \| D014635 \| YTHDC1 \| decreases \| \| Vorinostat \| D000077337 \| YTHDC1 \| decreases \| \| Acetaminophen \| D000082 \| YTHDC2 \| decreases \| \| Antirheumatic Agents \| D018501 \| YTHDC2 \| decreases \| \| butyraldehyde \| C018475 \| YTHDC2 \| decreases \| \| Cadmium Chloride \| D019256 \| YTHDC2 \| decreases \| \| chloroacetaldehyde \| C004656 \| YTHDC2 \| decreases \| \| Cidofovir \| D000077404 \| YTHDC2 \| decreases \| \| Cisplatin \| D002945 \| YTHDC2 \| decreases \| \| Copper Sulfate \| D019327 \| YTHDC2 \| increases \| \| Cyclosporine \| D016572 \| YTHDC2 \| increases \| \| Demecolcine \| D003703 \| YTHDC2 \| decreases \| \| dicrotophos \| C000944 \| YTHDC2 \| decreases \| \| epigallocatechin gallate \| C045651 \| YTHDC2 \| decreases \| \| jinfukang \| C544151 \| YTHDC2 \| decreases \| \| Lactic Acid \| D019344 \| YTHDC2 \| decreases \| \| Methyl Methanesulfonate \| D008741 \| YTHDC2 \| increases \| \| Naphthoquinones \| D009285 \| YTHDC2 \| increases \| \| Nickel \| D009532 \| YTHDC2 \| increases \| \| Oxygen \| D010100 \| YTHDC2 \| decreases \| \| perfluorooctane sulfonic acid \| C076994 \| YTHDC2 \| decreases \| \| Plant Extracts \| D010936 \| YTHDC2 \| increases \| \| potassium chromate(VI) \| C027373 \| YTHDC2 \| decreases \| \| Progesterone \| D011374 \| YTHDC2 \| increases \| \| Vincristine \| D014750 \| YTHDC2 \| decreases \| \| Acetaminophen \| D000082 \| YTHDF1 \| increases \| \| Atrazine \| D001280 \| YTHDF1 \| decreases \| \| dicrotophos \| C000944 \| YTHDF1 \| increases \| \| epigallocatechin gallate \| C045651 \| YTHDF1 \| increases \| \| Hydralazine \| D006830 \| YTHDF1 \| increases \| \| ICG 001 \| C492448 \| YTHDF1 \| increases \| \| Metribolone \| D015741 \| YTHDF1 \| increases \| \| Plant Oils \| D010938 \| YTHDF1 \| increases \| \| potassium chromate(VI) \| C027373 \| YTHDF1 \| increases \| \| Sunitinib \| D000077210 \| YTHDF1 \| increases \| \| Tobacco Smoke Pollution \| D014028 \| YTHDF1 \| increases \| \| Urethane \| D014520 \| YTHDF1 \| increases \| \| Valproic Acid \| D014635 \| YTHDF1 \| increases \| \| 1-Methyl-3-isobutylxanthine \| D015056 \| YTHDF2 \| increases \| \| 3-(2-hydroxy-4-(2-methylnonan-2-yl)phenyl)cyclohexan-1-ol \| C572491 \| YTHDF2 \| decreases \| \| 4-phenylbutyric acid \| C075773 \| YTHDF2 \| decreases \| \| 7,8-Dihydro-7,8-dihydroxybenzo(a)pyrene 9,10-oxide \| D015123 \| YTHDF2 \| decreases \| \| bisphenol A \| C006780 \| YTHDF2 \| increases \| \| C646 compound \| C584509 \| YTHDF2 \| decreases \| \| Copper Sulfate \| D019327 \| YTHDF2 \| increases \| \| Dexamethasone \| D003907 \| YTHDF2 \| increases \| \| ICG 001 \| C492448 \| YTHDF2 \| decreases \| \| Indomethacin \| D007213 \| YTHDF2 \| decreases \| \| Indomethacin \| D007213 \| YTHDF2 \| increases \| \| Methotrexate \| D008727 \| YTHDF2 \| decreases \| \| N(6)-methyladenosine \| C010223 \| YTHDF2 \| decreases \| \| Tobacco Smoke Pollution \| D014028 \| YTHDF2 \| increases \| \| (4-amino-1,4-dihydro-3-(2-pyridyl)-5-thioxo-1,2,4-triazole)copper(II) \| C517041 \| YTHDF3 \| increases \| \| 7,8-Dihydro-7,8-dihydroxybenzo(a)pyrene 9,10-oxide \| D015123 \| YTHDF3 \| decreases \| \| bicalutamide \| C053541 \| YTHDF3 \| increases \| \| C646 compound \| C584509 \| YTHDF3 \| decreases \| \| Cadmium \| D002104 \| YTHDF3 \| increases \| \| Copper Sulfate \| D019327 \| YTHDF3 \| increases \| \| Cyclosporine \| D016572 \| YTHDF3 \| decreases \| \| Demecolcine \| D003703 \| YTHDF3 \| increases \| \| Formaldehyde \| D005557 \| YTHDF3 \| increases \| \| Indomethacin \| D007213 \| YTHDF3 \| decreases \| \| jinfukang \| C544151 \| YTHDF3 \| decreases \| \| Plant Extracts \| D010936 \| YTHDF3 \| increases \| \| Plant Oils \| D010938 \| YTHDF3 \| increases \| \| Tobacco Smoke Pollution \| D014028 \| YTHDF3 \| increases \| \| Urethane \| D014520 \| YTHDF3 \| increases \| \| Vincristine \| D014750 \| YTHDF3 \| increases \| \| Zoledronic Acid \| D000077211 \| YTHDF3 \| increases \| |
